# Two sides of the same leader: an agent-based model to analyze the effect of ambivalent opinion leaders in social networks

**DOI:** 10.1007/s42001-022-00161-z

**Published:** 2022-04-26

**Authors:** Daniel Röchert, Manuel Cargnino, German Neubaum

**Affiliations:** University of Duisburg-Essen, Duisburg, Germany

**Keywords:** Opinion leader, Agent-based modeling, Ambivalence, Simulation, Network analysis

## Abstract

Opinion leaders (OLs) are becoming increasingly relevant on social networking sites as their visibility can help to shape their followers’ attitudes toward a variety of issues. While earlier research provided initial evidence on the effect of OLs using agent-based modeling, it remains unclear how OLs affect their network environment and, therefore, the opinion climate when: (a) they publicly hold ambivalent attitudes, and (b) they not only express support for their own stance but also discredit or ‘debunk’ the opposing side. This paper presents an agent-based model that determines the influence of OLs in social networks in relation to ambivalence and discreditation. The model draws on theoretical foundations of OLs as well as attitudinal ambivalence and was implemented using two network topologies. Results indicate that OLs have significant influence on the opinion climate and that an unequal number of OLs of different opinion camps lead to an imbalance in the opinion climate only in certain situations. Furthermore, OLs can dominate the opinion climate and turn their stance into a majority opinion more effectively when discrediting the opposing side. Ambivalent OLs, on the other hand, can contribute to greater balance in the opinion climate. These findings provide a more nuanced analysis of OLs in social networks by pointing to potential amplifications as well as boundaries of their influence. Implications are discussed with a focus on human and artificial key actors in online networks and their efficacy therein.

**Supplementary Information:**

The online version contains supplementary material available at 10.1007/s42001-022-00161-z.

## Introduction

The rise of digital communication platforms such as social networking sites has been posing new challenges in terms of understanding processes of opinion formation. The connectedness between users and the exchange of political information provides vast possibilities of mutual social influence. On social networking sites, opinion leaders (OLs) play a key role, as they can have a disproportionate influence on the opinions in their environments and thus the prevailing opinion climate. OLs are characterized by their high connectedness in networks, which enables them to steer the diffusion of information in a certain direction [1, 2]. However, on social networking sites, the influence of OLs is embedded in complex communication settings. For instance, OLs and other individuals do not always hold opinions that clearly favor one side. Instead, they can hold and express ambivalent opinions that equally favor both sides [3]. When it comes to OLs, public service broadcasters and non-partisan journalists may be considered ambivalent as they are bound to balanced reporting. Furthermore, public opinion or a network as a whole can be ambivalent when opinions on a political question vary between citizens (“network ambivalence” [4]). However, previous research has conceptualized opinion dynamics in social networks as the likelihood of expressing the valence of one’s opinion (i.e., to be in favor *or* against a political decision) without accounting for compelling social psychological evidence that indicates that individuals’ opinions are often not purely supporting or opposing an issue but can be ambivalent [5]–[7]. On social networking sites, the factual argumentation of statements is not always in the foreground, but instead, vulgarities and "dirty tricks" oftentimes characterize communication [8]. Public advocates of one viewpoint do not only talk about their stance, but also make references to “the other side,” be it in the form of counterarguing, providing substantial arguments, or even discrediting the credibility (e.g., the expertise and trustworthiness) of opponents and their views [9].

These two observations (i.e., the ambivalent and discrediting expressions of members of a network) have implications for how opinion climates evolve on social networking sites. Despite growing knowledge gained through agent-based modeling on the mechanisms that drive changes in opinion climates [10, 11], the observation that opinions (even those propagated by OLs) can be ambivalent or that key agents can discredit “the other side” have not been implemented in agent-based modeling to date. Taking these into account appears to be of pivotal relevance when it comes to explaining complex dynamics in online discussion networks. OLs may not necessarily advocate a clear and one-sided stance (e.g., spreaders of partisan media content), but instead convey equilibrated, i.e., ambivalent stances (e.g., spreaders of mainstream media or public service broadcasters). Including these aspects in the simulation of opinion formation processes allows for the modeling of more complex and realistic processes in large social networks. To our knowledge, there are no agent-based models to date in which attitude ambivalence is applied to OLs and users in networks. Agent-based models are best suitable to address social phenomena by simulating individual behavior and observing its impact on a group/network level [12].

Against this background, the present study is intended to use virtual simulations to: (1) include ambivalent opinions in the modeling of complex social influence processes in social networks; (2) implement ambivalence also in OLs’ expression behavior; and (3) take into account not only supportive expressions of viewpoints by OLs, but also discrediting utterances.

With that said, our work first acknowledges that OLs can be present simultaneously for different opinion camps. Still, it is yet to be understood whether variations in the number of OLs in different factions can make a difference in the ultimate outcome of the opinion climate. Therefore, we ask:

**RQ 1:** How does the opinion climate respond to a varying ratio of OLs in each group?

Second, we are interested in testing the effects of OLs on their network when they not only express one exclusive stance but also convey ambivalence in their utterances. While related work has shown that a like-minded social network environment can lead to a strengthening of users’ opinions [13] and underlines the important role of OLs in the process of diffusion [14], little is known on the impact of ambivalent opinions conveyed by OLs. Therefore, we aim to compare effects of univalent and ambivalent OLs on the network level and ask:

**RQ 2:** How does the opinion climate respond to OLs who advocate fully in favor of their stance, are fully ambivalent, or partly in favor of the opponents’ stance?

Third, we also intend to examine the situation in which an OL not only presents support for a stance but also actively discredits or debunks the opposing side:

**RQ3:** How does the opinion climate respond to OLs of one side who advocate fully in favor of their stance and discredit the opponents’ stance?

## Theoretical background

In this section, we outline the theoretical background of OLs and how they have already been implemented in agent-based models. Furthermore, we discuss the theory behind attitudinal ambivalence as well as how related psychological processes can be applied to the model of OLs.

### Opinion leaders in social networks

The networking of individuals in social networking sites facilitates not only rapid communication among users, but also exposure to a variety of information and different opinions. OLs can play a key role in this process, as they are not only connected to numerous users in the network, but also have a strong influence on other users’ opinions. “Generally, opinion leader is obviously the critical node with a higher centrality in social networks, and it is bound to affect public opinion assimilation, integration, and separation, but his/her influence on opinion evolution is obviously different from that of ordinary individual […]” [15, p. 3]. Lazarsfeld et al. [16] introduced the notion of OLs while studying US presidential elections. They investigated electoral behavior and proposed a two-step flow model: mass media indirectly influence the general public by first providing information to OLs who, in turn, pass this information on to individuals [16, 17].

Due to the fact that the use of social networking sites has been increasing in recent decades, research has started to study how OLs and their influence manifest themselves in social networking platforms. Studies have shown that OLs play a major and central role in the dissemination of information in online discussions [14], having influence on individuals [18] even if they are politically uninterested [19]. A recent study based on the social media debate on climate change showed that political actors are highly qualified to fulfill the position of OLs, as they have a significant impact on the flow of information, as characterized by the fact that they posted a higher volume of tweets and were also more frequently mentioned by other users [20].

The concept of OLs is often associated with information diffusion, which describes the process of how information is spread within the network. Based on results focusing on OLs and diffusion processes, research showed that the presence of only a few OLs can have an impact on how quickly information is spread in online networks [21, 22]. Simulation studies have identified that OLs with high “sociality” (i.e., the total strength of ties of an entity) are best placed to rapidly disseminate information. However, OLs only influence the diffusion process if the percentage of first-time addressees reaches a critical mass [22]. Previous studies have shown that the proportion of OLs varies and depends on the object of investigation. While Choi [23] identified a proportion of only 4% of OLs in a study on Twitter-based discussion groups in South Korea, previous surveys on offline OLs identified 23%–30% of respondents as OLs [24]. A similar distribution was also used in an agent-based simulation [25, p. 201]. Finally, a detailed examination by Weeks [2] identified 12.5% of users as OLs.

While these findings imply that the number of OLs varies, little is known on the impact that different proportions of OLs might have on the dynamics of opinion formation and resulting opinion climates in online networks.

#### Influence: conceptual issues

Related work on processes of opinion formation and influence through influential players (i.e., OLs) used different concepts and operationalizations. For instance, Bakshy et al. [26] studied influence among Twitter users based on network diffusion. Accordingly, influence is understood as the degree to which a piece of information is spread through the network. The wider a piece of information is spread (i.e., the larger the size of the “diffusion tree”), the more influential its sender. According to this view, influence is something that can be directly observed through the extent of information diffusion [20], which makes OLs influential players, since information stemming from them is of relatively high reach. The present work addresses influence mainly on the level of opinions or attitudes toward a specific issue (e.g., a controversial policy), i.e., on the level of internal psychological processes. On the network level, influence is thus characterized by the degree to which a user’s opinion is impacted by opinions represented in their environment. Consequently, OLs are influential, since opinions propagated by them have a relatively high impact on neighboring opinions [2]. This concept of influence most closely resembles models of opinion formation (in particular, the two-step flow model, see [13, 14, 22]) and psychological models of social influence (e.g., [27, 28]), which refer to the role of influential single actors (i.e., OLs) and groups (e.g., a local environment within a social network) in processes of opinion formation, respectively.

### Agent-based modeling of opinion dynamics

With a focus on the actual communication process, there is already a vast body of research that employs agent-based modeling to address the dynamics of opinion formation processes in social networks [29, 30]. The use of agent-based modeling offers the advantage that models can be used to simulate “how individuals and the environmental variables influencing them vary over space, time or other dimensions” [31, p. 11]. Thus, by generating models that represent agents and their interactions with a certain phenomenon under realistic conditions, it is possible to test even those theories that could otherwise only be addressed with very extensive empirical studies in which social interactions are observed in the long run [32]–[34]. It has been noted that there is a gap between the micro- (i.e., individual actions) and macro- (i.e., societal dynamics) level when it comes to investigating social phenomena [32, 35, 36].

Agent-based modeling studies also demonstrated the influence of OLs on the formation of opinions of individuals in their networks [25, 37, 38]. In a recent agent-based model, Borowski and colleagues [37] showed that the number of OLs and their ability to maximize information diffusion depends solely on the network structure. These results are in line with the study by van Eck and colleagues [25], who analyzed the influence of OLs and demonstrated that the velocity at which information is transmitted depends strongly on the network position of the OLs.

While these studies have corroborated the key role OLs play in social networks, they all modeled OLs as advocates for a certain stance who, in turn, were embedded in networks in which individuals were either supporting or opposing a political stance. However, social psychological research has consistently shown that holding an opinion on a political question can be more complex than just assuming a pro or contra stance [5]–[7].

### Attitudinal ambivalence

Intuitively, most individuals would likely describe an opinion as something that can be roughly divided into the dichotomy of ‘in favor’ and ‘against.’ Similarly, research into political attitudes has, more or less implicitly, been conceiving of attitudes as one-dimensional constructs and measured them on scales that usually range between the two poles of ‘completely against’ and ‘completely in favor.’ One problem with this type of measurement is that responses often scatter closely around the scale's midpoint, and researchers have been interpreting this as a ‘neutral’ attitude [3, 39]. However, this interpretation may not be valid, since attitudes are oftentimes more complex. The concept of ‘attitudinal ambivalence’ accounts for this complexity and describes attitudes as two-dimensional, i.e., it does not conceive of positive and negative evaluations of an attitudinal object as endpoints of one and the same continuum, but instead as two independent dimensions [3, 6, 40].

Accordingly, an ambivalent attitude simultaneously entails favorable and unfavorable evaluations toward an attitudinal object. As a consequence, a response on the scale midpoint may not only indicate neutrality, but instead be the result of an ‘internal averaging’ of the positive and negative evaluations [39]. Ambivalent attitudes are different from neutral attitudes or indifference (i.e., weak attitudes), since they entail equally strong evaluations of opposing poles [3]. In line with this, Thompson and colleagues [40] characterize ambivalent attitudes as being linked to equally strong positive and negative evaluations of at least moderate size. To determine the degree of ambivalence then, one simply needs to subtract the two attitude components from each other: the closer the resulting value is to 0, the higher the ambivalence toward the object (for a similar procedure, see [41]). Even though the inconsistency induced by ambivalence can be linked to aversive affective states in some cases [42], it is likely that ambivalent attitudes are, in general, very common: they have been found with regard to many different attitudinal objects, among them political issues (see [3, 43]). However, due to the widespread use of one-dimensional attitude conceptions in research on political opinions, they have likely been neglected in much of the extant work [39]. Ambivalent attitudes can promote conflicting intentions and thereby undermine the execution of behaviors linked to the attitudinal object (e.g., voicing an opinion in public; [44, 45]).

When it comes to discussions on social networking sites, those users who have an ambivalent attitude toward an issue may hence be less likely to express a clear stance toward that issue (and more likely to express balanced views). At the same time, ambivalence may result from exposure to political information in the first place [39, 45]. For instance, an individual might have a non-ambivalent attitude toward the COVID-19 policies but then get exposed to information supporting the contrary, thereby changing the overall evaluation of COVID-19 policies toward an ambivalent attitude. In short, attitudinal ambivalence can both shape the structure of an online discussion network and result from network effects. The present study takes both aspects into account and addresses the dynamics of mutual social influence in discussion networks that include the expression of ambivalent attitudes.

#### Psychological processes underlying attitudinal ambivalence

The observation that people’s attitudes do not always fit into a unidimensional framework in the sense of being exclusively in favor or fully against something raises the question of how the prevalence of attitudinal ambivalence affects social influence dynamics in public opinion [4]. Being exposed to critical claims that oppose one’s own is key to building mutual understanding and shared knowledge, as well as to fostering education for the effective functioning of democratic systems [46]. Following this logic, ambivalence may be desirable not only on a collective (i.e., an ambivalent opinion climate among citizens) but also on an individual level (e.g., a person holding diametrical views on a certain political decision). Therefore, the present work focuses on the situation of being exposed to ambivalent rather than uniformly opposing views, the effects of which on political communication behavior is yet to be examined.

The missing link between ambivalence and political behavior prompts research to shed light on how attitudinal ambivalence manifests itself. Following the notion of the value pluralism model [47], scholars have proposed that attitudinal ambivalence should be associated with an “integrative complexity” or “balanced judgment,” that is, individuals are capable of evaluating issues based on diverse and even contradictory information [48, 49]. While this state of ambivalence was found to evoke more thorough processing of newly incoming information [41, 50], ambivalent attitudes are more likely to increase uncertainty and induce more moderate attitudes [49]. Visser and Mirabile [51] demonstrated that people to whom individuals are directly connected, that is, in terms of their social networks, can be responsible for an individual’s increasing attitudinal ambivalence. They argue that individuals compare their own attitudes with those of people around them and, in cases where they assess a conflict, they experience not only an intrapsychic tension due to the conflicting attitudes, but also “interpersonal conflictive tension” due to the connection to the person. Their findings show that attitudinally heterogeneous social networks (i.e., networks consisting of people with whom one agrees but also disagrees) foster individuals’ attitudinal ambivalence and, therefore, decrease the strength of these attitudes. The power of the social environment and its influence on people’s attitude, strength, and ambivalence has been emphasized and revealed by further studies [39, 52]. However, it remained unclear what role univalent and ambivalent key actors in networks—OLs—might play in the social influence process. So far, work on the effectiveness of influencers suggested that influential players in the network can have a significant impact on other users [53].

## Method

Based on the outlined theoretical foundations and empirical evidence, we developed an agent-based model to examine our research questions (model, data, and results can be found in the repository of the Open Science Framework). For the implementation, we used NetLogo [54], which works in conjunction with the package RNetLogo [55].

### Opinion domain

There are numerous models regarding the investigation of the evolution of opinions, and these models can be differentiated between discrete (Voter model: Clifford & Sudbury [56], Holley & Liggett [57], Sznajd model [58]) and continuous models (DeGroot [59], Deffuant-Weisbuch [60], Hegselmann-Krause [61]). While in the discrete models, the agents' value space is binary, in continuous models, it can be in a continuous value interval. In continuous models, we can distinguish between those in which agents have their opinions influenced based on like-minded neighbors (bounded confidence) and those in which opinions are updated based on a weighted average of neighbor’s opinions. In addition to these classical models, there are specific models that, although not developed primarily for opinion dynamics processes, are still suitable for this kind of dynamics process. The SIR (Susceptible, Infectious, or Recovered) model was first used to forecast the spread of diseases based on mathematical equations [62] and was also applied to opinion dynamics problems [63–65]. However, due to the different phases of this model, problems arise in the opinion consensus of the group, which is why further models for opinion dynamics processes were developed [66]. Furthermore, it was found that individual key nodes with a higher degree do not have a higher influence on the neighboring nodes [67]; these effects make the SIR model not further applicable for our consideration of OLs.

**Fig. 1:**
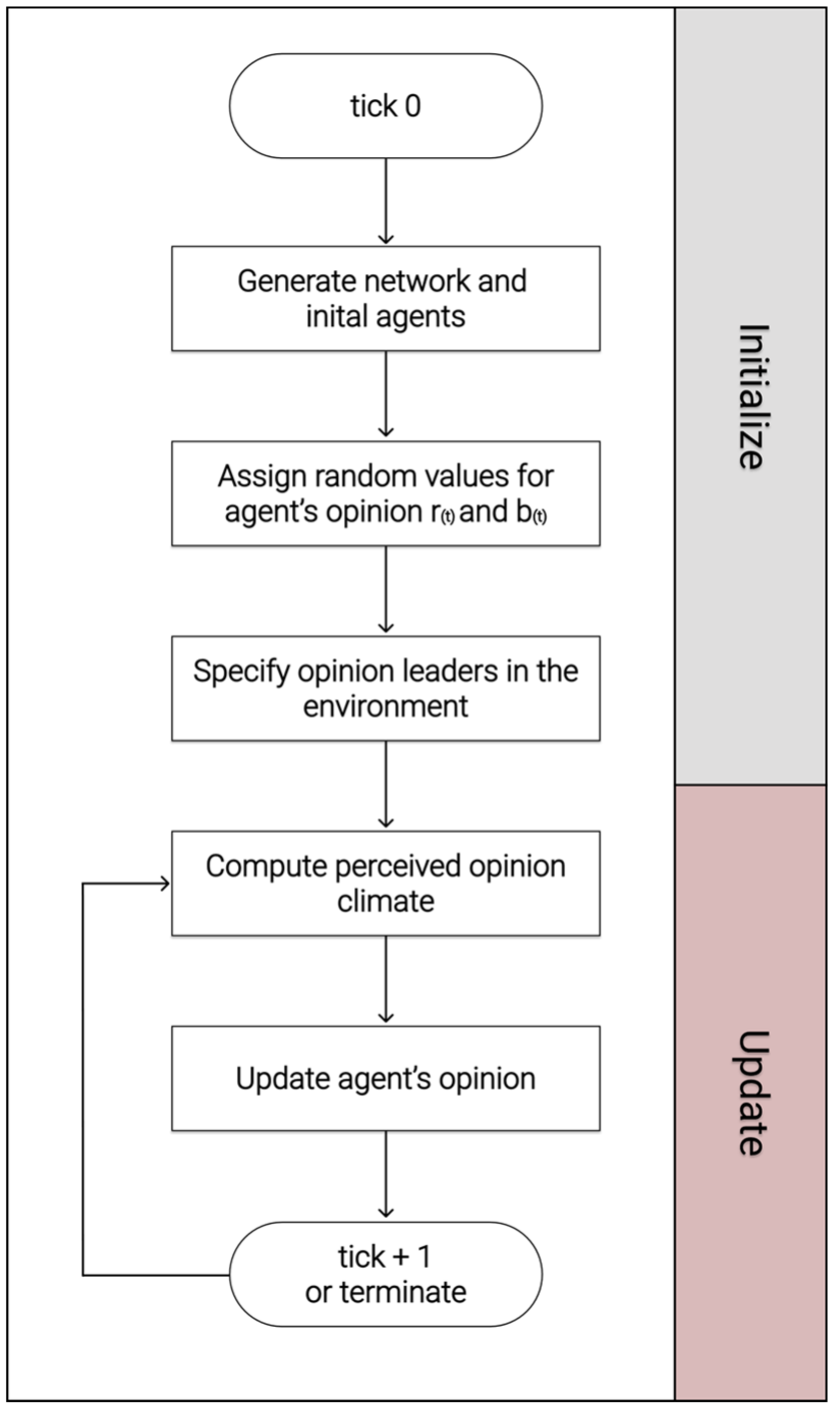
Graphical representation of the model and its functionalities

Our model, as specified below, is related to the DeGroot model, which follows the principle of social influence and assumes that the adjacent neighbors of an individual have an influence on opinion formation. In general, the DeGroot model implies that the agents strive for a common consensus, which is mainly achieved by the individual weighting of the agents, where this is constant and thus static over the entire course of the process. In the DeGroot model, the individual's updated opinion is simultaneously determined based on the confidence weights of the edges in the network and thus as a weighted average of their own current opinion and that of their neighbors [59]. One of the reasons we decided to focus on the DeGroot model is that it has been already used for numerous studies in the field of opinion dynamic due to its simple mechanism of updating opinions and its ease of extension, which allows researchers to customize the model according to their individual circumstances and specifically to their object of study. In our case, using the DeGroot model, we can directly represent the social influence of an agent in its network environment by measuring the perceived opinion climate of nodes based on their connected neighbors to update the opinions of the agent in a two-dimensional spectrum. Therefore, the use of the DeGroot model fits well to answer our research questions, as social influence and the related opinion dynamics can be considered in a social network. This is based on an iterative averaging model, in which agents' opinions are linked to neighboring nodes and thus considered to determine a kind of "opinion climate." This aspect of social influence is especially relevant for opinion leaders, as they operate in social network structures and can influence followers through their opinions. Since the DeGroot model is based on graph theory, the connections of nodes are enabled by means of edges and can be used for complex computations. Just as important as in the DeGroot model, the consideration of opinions from neighboring nodes has a major contribution in our implementation of the ABM to compute the perceived opinion climate and determine their update function. However, there are two major dissimilarities from the original DeGroot model that are manifested in our model. In our model, we have extended the DeGroot model to a two-dimensional opinion spectrum (an agent has a red opinion and a blue opinion), which are in competition with each other and allow agents to exhibit ambivalent behavior. By realizing the two-dimensional opinion observation, more complex mechanisms can be captured. Previous studies have also shown that the results from a two-dimensional opinion range lead to the same results as in a one-dimensional model [68, 69] Therefore, we assume that our results of a two-dimensional vector compared to a one-dimensional vector might be similar for the first research question. Another difference to the original DeGroot model is the update function. Since this is static in the initial DeGroot model and does not change for the individual agents, we have introduced a dynamic gradation which depends on the individual opinion of the agent and the strength of the perceived opinion climate. This update function is applied to the two-dimensional opinion image of the individual agents and can change continuously as it progresses through the perceived opinion climate. To represent a detailed and transparent illustration of our model, we have depicted the sequential flow of our applied model in Fig. 1 below.

Figure 1 can be viewed in combination with the pseudocode from Table 1, which is responsible for the process of initializing the model, and Table 2, which contains the update function of the model. The graphical representation as well as the pseudocode provide a deeper understanding to provide a more comprehensive understanding of the processes and their functionality. In the further course of the paper, the following sections implicitly refer to the description and explanation of the individual processes and how they are defined in detail.

**Table 1:**
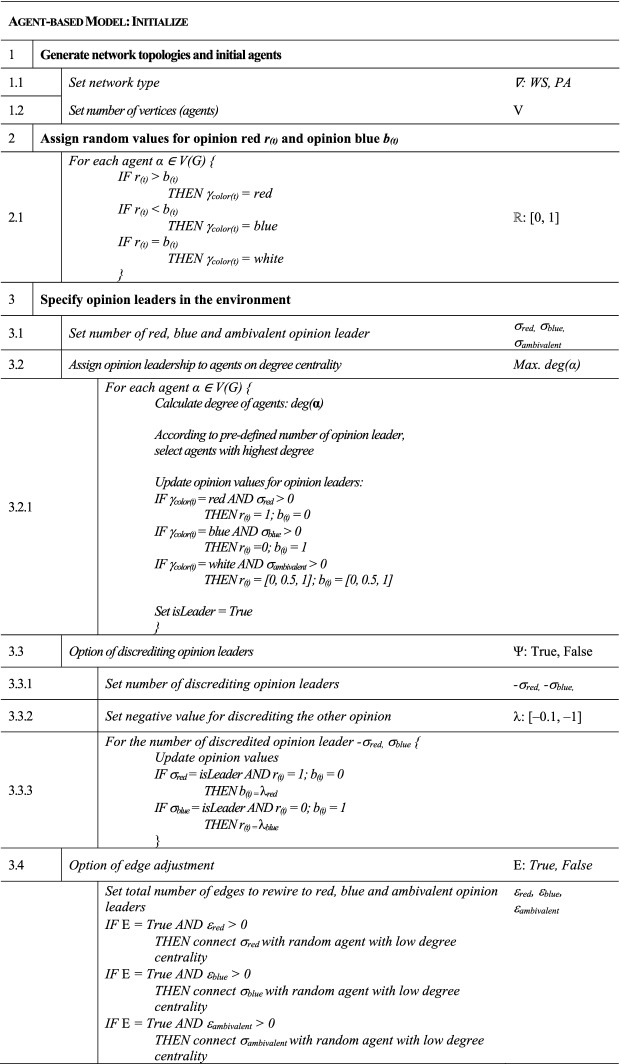
Mechanism and sequence of initialization of the model in pseudocode

**Table 2:**
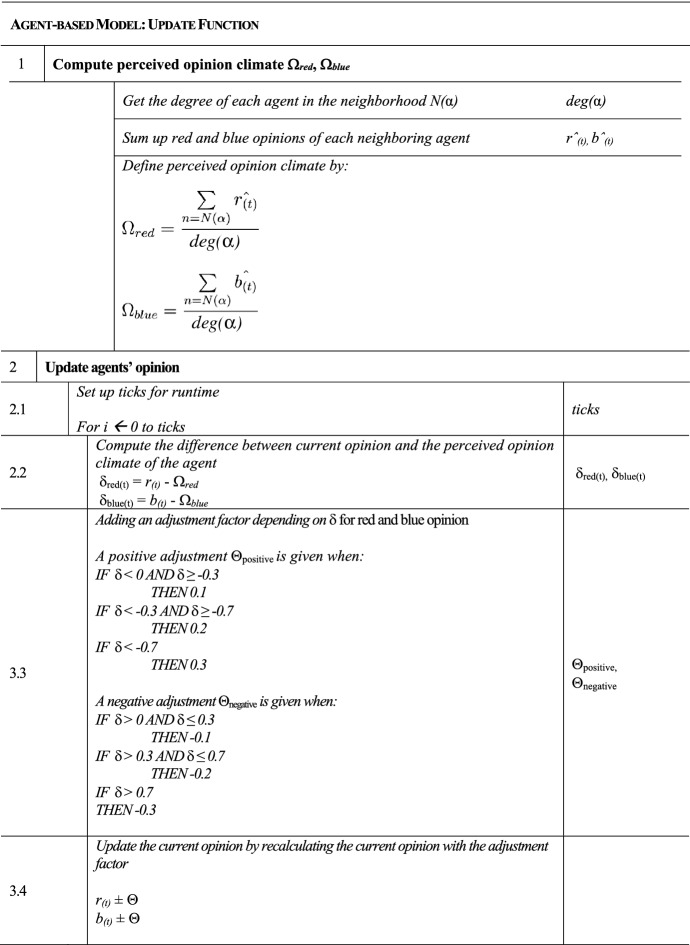
Mechanism and sequence of the update function of the model in pseudocode

### Interaction direction and symmetry

We decided to represent the connection between individual agents and OLs in a network topology to guarantee the flow of opinions and their communication landscape. This means that the modeling is based on the principles of graph theory, where a graph $G$ is defined as an ordered pair $G = (V,E)$, where $V$ is a set of agents and $E$ is a set of edges. We have adopted an undirected network, as this allows a bidirectional communication flow between agents, thereby ensuring that the agents not only express their own opinion but also come into direct contact with other opinions (thus resembling communication processes on social networking sites). Based on the theoretical background outlined earlier, we opted to apply two types of network topologies ∇ for our modeling: (a) the Barabási–Albert preferential attachment model [70] and (b) the Watts–Strogatz model [71]. Including both network topologies allowed us to test the robustness of the individual network structures. Since we build on a two-dimensional opinion model in our setup, we do not predict specific weights of the edges, but have used a predefined scheme to determine different thresholds that symmetrically update and adjust the two different opinions in the network.

#### Network models according to Barabási–Albert and Watts–Strogatz

Our investigated network structures have already been used in the literature based on opinion dynamics to explain specific use cases or social phenomena [34, 72]–[74]. In addition, the character properties emanating from the two network topologies are a crucial reason why we implemented them in our modeling in the context of opinion leadership. Here, the power law property is a key point, which is found not only in scale-free networks but has also been demonstrated in real social networks [75]. It was found that this distribution exists in both YouTube [76, 77] and Facebook [78] networks, where some users were also characterized with a very high degree. Based on our definition of OLs, we assume that social networking sites such as YouTube and Facebook provide a means for individuals to get in touch with other people and exchange opinions among themselves (for example, in the form of comments). In this process, OLs that have a significant impact on and influence the opinions of other users in the network can emerge. Furthermore, since this aspect in particular reflects the preferential attachment model, we decided to include the Watts–Strogatz model as a comparison in our analysis. Both network topologies are well-established models in different fields of science to explain complex structures and dynamic processes of networks in the real world and still have significant relevance to expand the understanding of network science today [79]. While the Watts–Strogatz model is based on a kind of friendship network, where friends are connected to other friends (clustered connectivity), the Preferential Attachment Model aims rather at the formation of individual hubs, which have more relationships to other nodes because they appear more attractive (heterogeneous connectivity). Furthermore, Hein et al. point out that network topologies may have an influence on the outcome of simulation studies, which makes it even more important to investigate different topologies [80]. Accordingly, examining these two network topologies in relation to opinion leaders might reveal a way to infer statements about opinion dynamics and their influence on neighboring agents.

The Watts–Strogatz model is a randomized network belonging to the family of small-world networks that is more common in reality and is characterized by properties such as high clustering coefficients and short average path length [71]. Studies that have looked at information dissemination have also found that Watts–Strogatz networks perform similarly to scale-free networks [81, 82]. For these aforementioned reasons and our definition of OLs, we decided to use these two network topologies in the further course of our modeling. We describe the individual network models in more detail below:

The preferential attachment model by Barabási and Albert produces networks that are scale-free, i.e., have a power law degree distribution [70]. The actual functionality of network generation is that new nodes are preferentially connected to nodes with a high degree of connectivity, which ensures that an older node has many connections. Scale-free networks are based on the principle of preferential attachment and thus automatically provide a dynamic network structure, i.e., the addition of new nodes to an already well-connected node [37]. The equation of the preferential mechanism is defined by

$$P(k_{i} ) = \frac{{k_{i} }}{{\sum\limits_{j} {k_{j} } }}$$

where $P$ is the probability to link a newly connected node to node $i$, which is dependent on the degree $ki$ of node $i$. This mechanism results in a power law distribution and thus the principle "the rich get richer,” where nodes with a high degree are preferred. Due to its natural ability to generate networks with power-level degree distributions, the Barabási–Albert model is generally deemed a good choice for modeling social networks.

#### Rewiring edges

**Fig. 2:**
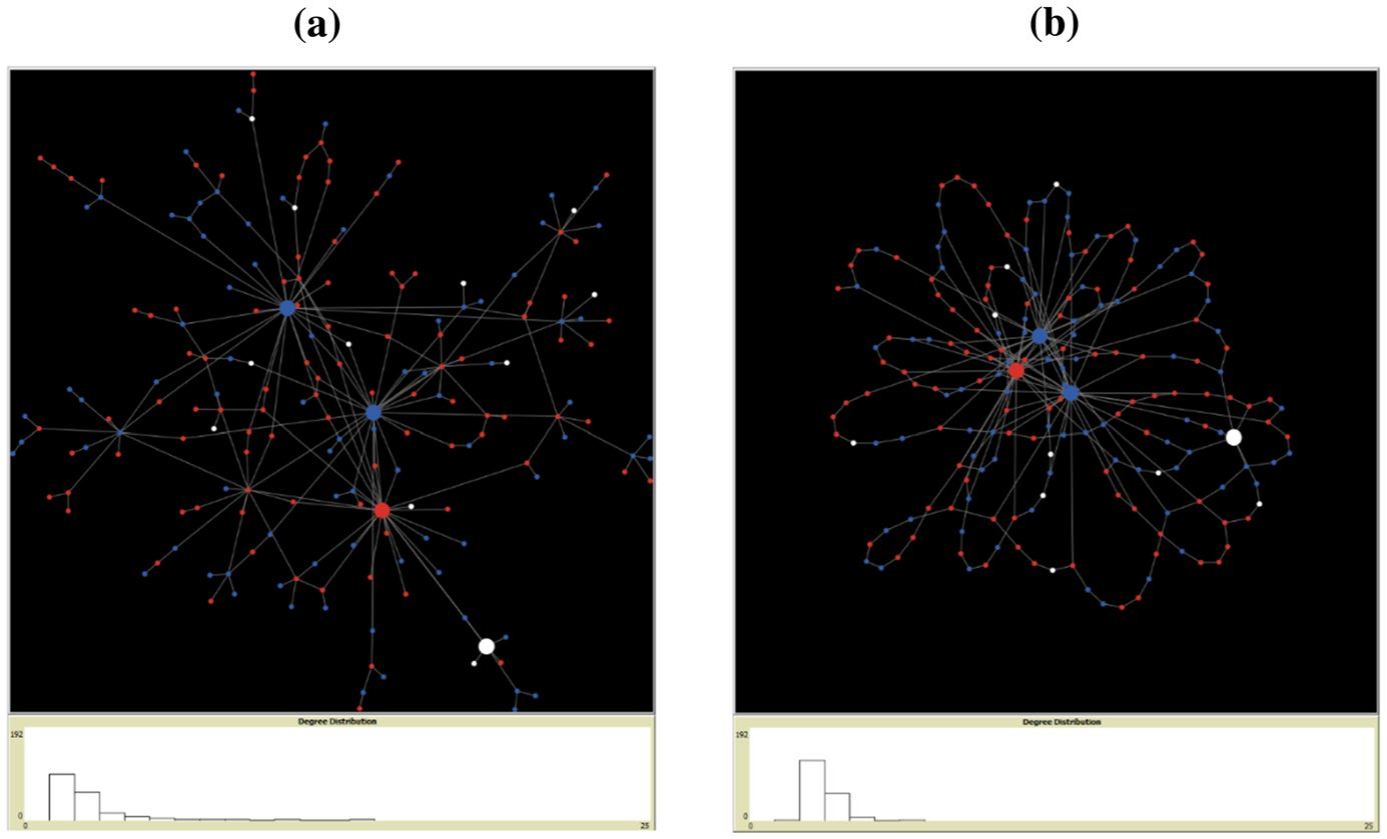
Network topologies: (**a**) Barabási–Albert, (**b**) Watts–Strogatz

To ensure that we fitted the definitions of OLs with numerous connections and greater influence on the opinion climate in the network, we decided to consider and apply the basic idea of randomly adding further edges to random OLs after the network had been created, whereby a low degree value was observed. The parameter ($\epsilon$) can be enabled or disabled and assigns the number of edges that randomly connect from an agent to a random OL (${\epsilon }_{blue}$,${\epsilon }_{red}$,${\epsilon }_{ambivalent}$). This rewiring makes it possible to create different distributions of OLs, where, for example, one faction has fewer OLs, but is very strongly connected, while the other faction is less strongly connected but has more OLs. Figure 2 shows the generated network topologies with connected agents and OLs.

### Interacting agents

In our model, each agent $\alpha$ holds a two-dimensional opinion, i.e., a “red” opinion ${r}_{(t)}$, and a “blue” opinion ${b}_{(t)}$, which represents a stance contrary to ${r}_{(t)}$ on the same issue. Opinions are represented by real values in the [0, 1] interval and change in several time intervals $t$. Values represent the extent to which a person is for (*r*) or against (*b*) a certain issue. It is important that this value is randomly assigned between 0 and 1, so that no bias occurs. To avoid very long decimal numbers, we round the value to the first decimal place. The value between 0 and 1 can be interpreted to some extent as possible arguments for the respective opinion. More precisely, if $\alpha$ has the initialization value of ${\mathrm{r}}_{(0)}= 0.4$ and ${b}_{(0)}= 0.2$, it could be interpreted that it has four arguments in favor of the red stance, while it has only two arguments favoring the blue stance. These two values determine the attitude of each agent for the classification in a certain opinion camp as well as for the presentation in the network.

Thus, if the value of ${r}_{(t)}$ is higher than the value of ${b}_{(t)}$, this means that there are more arguments from the red opinion than from the blue opinion, and thus, the agent is inclined to favor the red opinion. If both opinions hold the same value, the agent holds an ambivalent opinion and is marked as a white node in the network. The color ${\gamma }_{{color}_{(t)}}$ represents the agent's opinions over time and is controlled in the model with the following rule:

$$\gamma_{{color_{(t)} }} = \left\{ \begin{array}{ll} red, &\quad if\ r_{(t)} > b_{(t)}\\ blue,&\quad if\ r_{(t)} < b_{(t)}\\ white,&\quad if\ r_{(t)} = b_{(t)} \end{array}\right.$$

These values constantly change in the course of the simulation and, therefore, the properties of the agent in the network also change.

To determine the perceived neighboring opinion climate $\Omega$ of connected nodes from $\alpha$, it is essential to identify $\widehat{{r}_{(t)}}$ and $\widehat{{b}_{(t)}}$ of the neighborhood $N()$. The neighborhood of an agent $\alpha \in V(G)$ is the set of all nodes that are adjacent to $\alpha$ and defined as $N(\alpha ) = \{y \in V(G) : \{\alpha ,y\} \in E(G)\}$. Starting with one agent, we calculate the neighborhood and determine the sum of $\widehat{{r}_{(t)}}$ and $\widehat{{b}_{(t)}}$ of all connected agents in the network. The sum of this is the perceived neighboring opinion climate of a single agent$\alpha$. To include the number of connected nodes in the calculation of the perceived neighboring opinion, this is divided by the degree of alpha $deg(\alpha )$. The degree is defined as the number of nodes adjacent to $\alpha$ and therefore the size of the neighborhood of $\alpha$, that is, $deg(\alpha ) = | N(\alpha ) |$. The following equations show the perceived neighboring opinion climate of a specific agent and their neighboring opinions $\widehat{{r}_{(t)}}$ and $\widehat{{b}_{(t)}}$:

$$\Omega_{red} = \frac{{\sum\limits_{n = N(\alpha )}^{{}} {\widehat{{r_{(t)} }}} }}{deg(\alpha )}$$

$$\Omega_{blue} = \frac{{\sum\limits_{n = N(\alpha )}^{{}} {\widehat{{b_{(t)} }}} }}{deg(\alpha )}$$

With the newly computed factor of the perceived neighboring opinion climate ${\Omega }_{red}$ and ${\Omega }_{blue}$, the difference ${\delta }_{red(t)}$ and ${\delta }_{blue(t)}$ to the outgoing single agent opinions ${r}_{(t)}$ and ${b}_{(t)}$ is then determined. This value is rounded to the third decimal place and allows for a more fine-grained view of opinions and the further course. This computation is necessary, since it ensures that the existing opinion climate of an agent is directly referenced with their own opinion, thus taking into account the effect of the network and its nodes.

$${\delta }_{red(t)} = r_{(t)} -{\Omega }_{red}$$

$${\delta }_{blue(t)}= b_{(t)}-{\Omega }_{blue}$$

#### Updating function

If the value of the differential is positive, a negative adjustment factor ${\Theta }_{negative}$ is added to the current opinion, while for a negative value of the differential, a positive adjustment factor ${\Theta }_{positive}$ is updated to the current opinion. Thus, we ensure with the following formulas, ${r}_{(t)}\pm \Theta$ and ${b}_{(t)}\pm \Theta$, that the two opinions are recalculated per tick to guarantee a constant adaptation of the model. Finally, we also round the value of the newly computed opinion to the second decimal place, since the implementation of an ambivalent opinion space (${r}_{(t)}{== b}_{(t)})$ is otherwise not feasible due to an extremely small probability and doing so also improves the performance within our model for the further course. We have chosen the following adjustment values $\Theta$ for specific intervals, so that even in a more strongly represented opinion climate, the adaptation of the individual agent manifests itself in a stronger form. To be more precise, if δ is greater than the agent's opinion, this means that the agent adapts their opinion to the climate of opinion. The stronger δ, and thus the perceived climate of opinion, the stronger the agent adapts to this opinion—and likewise, even if the perceived climate of opinion is represented as weaker than the current opinion. Here, the value of the opinions is then corrected downward, since the agent feels no influence of their environment.

$$ \Theta _{{positive}}  = \left\{ {\begin{array}{l}    {0.1,} \hfill  \\    {0.2,} \hfill  \\    {0.3,} \hfill  \\   \end{array} } \right.\begin{array}{l}    {if\;\;\delta  < 0\;and\;\delta  \ge  - 0.3} \hfill  \\    {if\;\;\delta  <  - 0.3\;and\;\delta  \ge  - 0.7} \hfill  \\    {if\;\;\delta  <  - 0.7} \hfill  \\   \end{array} $$

$$\Theta_{negative} = \left\{ {\begin{array}{l} { - 0.1,} \hfill \\ { - 0.2,} \hfill \\ { - 0.3,} \hfill \\ \end{array} } \right.\begin{array}{l} { if \;\;\delta > 0 \;and\; \delta \le 0.3} \hfill \\ { if \;\;\delta > 0.3 \;and\; \delta \le 0.7} \hfill \\ { if \;\;\delta > 0.7} \hfill \\ \end{array} $$

This approach to modeling conceives of influence as a linear function of opinions within the social environment, as found in various accounts of opinion formation and social influence [27, 28, 83] and as previously found in large-scale social network data [17]. While work on psychological reactance and so-called “backfire effects” [84, 85] would suggest a more complex process that includes the possibility of non-linear adaptions (e.g., opinion change in the opposite direction of opinions represented within the network environment), the present work models opinion dynamics based on accounts that are more parsimonious (yet empirically well-founded) and which allow our model to remain more simple.

### Interacting opinion leaders

In our model, OLs $\sigma$ have the same characteristics as other agents (i.e., users), but they differ in their position in the network and in the constant opinion values with which they influence the opinions of connected agents. Since the position of OLs is a key factor for the diffusion of information in the network [25], we adhered to the results of previous research in our modeling. There are different approaches in terms of centrality measures (degree, betweenness, and closeness) to determine these nodes of OLs in networks. Xiao and colleagues [15] also used an agent-based model to investigate the dynamic processes of OLs in networks and found that the detection of the three types of centralities (degree centrality, betweenness centrality, and closeness centrality) have a similar influence on opinion formation and thus differ only marginally. As previous research has demonstrated, OLs in networks are characterized by the fact that they hold a higher in-degree centrality in the network and therefore have more influence on individuals [15]. Other studies have also taken the measurement of degree centrality as a criterion for detecting OLs in real-world social networks ([86, 87]) and defined this as an indicator of local OLs [88, 89]. Once the network had been generated and each agent had been initialized, we characterized OLs on the basis of degree centrality. Degree centrality measures the number of connections of nodes connected to a particular node; the higher the degree centrality of a node, the more influence it has in the network. The calculation of degree centrality is defined as follows:

$$C_{Di} = \sum\limits_{j} a_{ij}$$

At this point, we only know which nodes have greater influence in the network; however, these OLs are not yet assigned to an opinion camp. The next step is to randomly assign the OLs; to ensure this, we compute the sum of the requested OLs across all opinion camps to filter and select only nodes characterized with the Nth highest degree centrality. Once we had identified the nodes with the highest degree centralities, the OLs were randomly assigned to the red and blue opinion according to the highest degree centrality of each node. The randomization process ensures that the distribution of the opinion camp is equitably distributed and that we do not create bias in the positioning of OLs in the network. This procedure also applies to the ambivalent OLs.

#### Univalent and ambivalent OLs

We distinguish between univalent and ambivalent OLs that are able to influence the opinions of the agents in the network. For the univalent OLs the values are different (i.e., ${\sigma }_{red}:$ *r* = 1 and *b* = 0, or, ${\sigma }_{blue}:$ *r* = 0 and *b* = 1) to model a strong univalent influence on the opinions. For ambivalent OLs, the values are identical and moderate (i.e., ${\sigma }_{ambivalent}:$ *r* = 0.5 and *b* = 0.5) or high (i.e., *r* = 1 and *b* = 1). Consequently, OLs can equally influence both opinions of the agents they are connected with. Additionally, our modeling allows us to vary the total number of OLs in the network and thus to split the distribution specifically. Different distributions of OLs can be simulated in the model with regard to the number of ambivalent, red, or blue OLs present in the network.

#### Discrediting OLs

In addition to the functionality of the OLs, we have also considered the case of OLs that discredit their opponent's position. The functionality of discrediting Ψ can be switched on and off depending on the configuration of the model, where on the one hand the quantity of discrediting OLs $-\sigma$ and on the other hand the intensity of the discrediting ${\lambda }$ toward the opposing opinion camp can be determined. The intensity of the discrediting ${\lambda }$ has been implemented in our model by a negative value of [–0.1 to –1] for the opposite opinion, so that, e.g., an OL in the red opinion camp has the values *r* = 1, *b* = ${\lambda }_{blue}$, ${\lambda }_{blue}$ = –0.5. The negative values have a direct influence on the temporal course of the modeling, since OLs in this case not only increase neighboring agents’ values with regard to one camp (e.g., ${r}_{(t)}$), but also decrease their values regarding the stance toward the opposing camp (e.g., ${b}_{(t)}$).

**Fig. 3:**
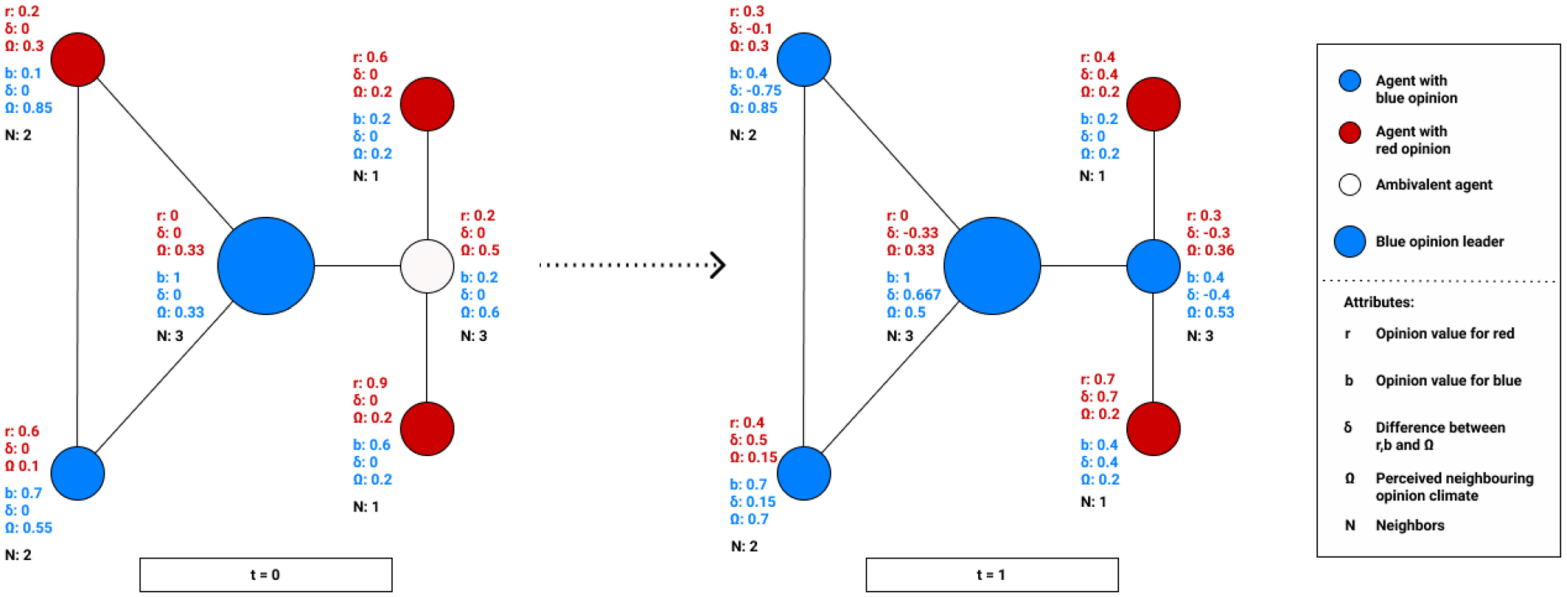
Example of an opinion update

Figure 3 shows the mechanism of the influence of an OL and how the opinion climate changes. It can be seen that the blue OL has a strong influence on the ambivalent node as well as on the directly connected red node. Consequently, the OL has succeeded in influencing agents in their direct environment toward a majority of blue nodes.

**Fig. 4:**
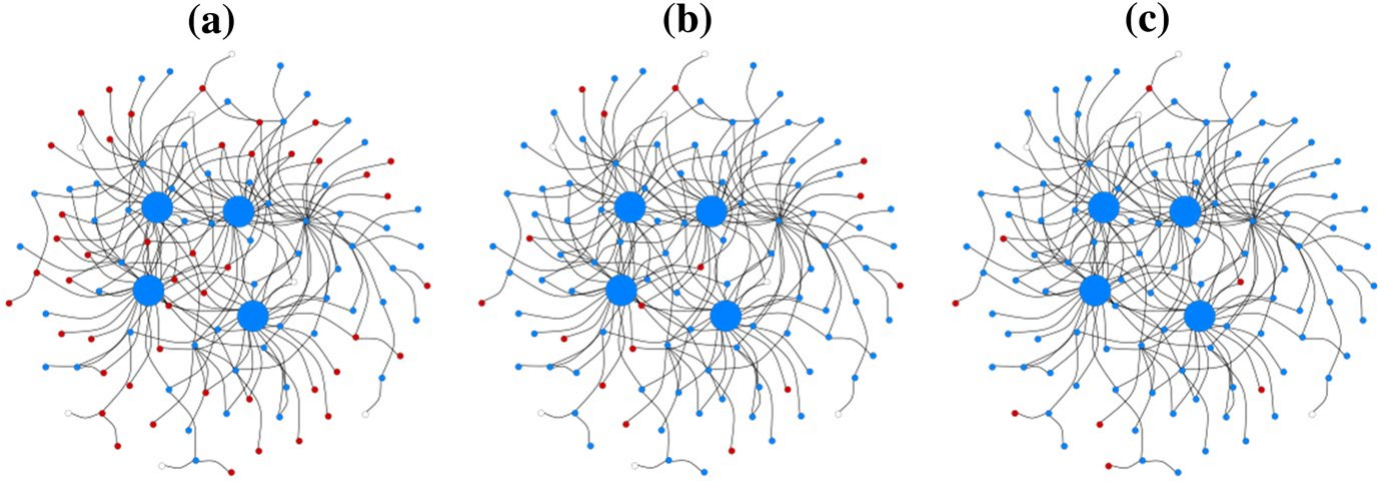
Example of opinion formation by four opinion leaders: (**a**) at the beginning with (${\upgamma }_{\mathrm{blue}}=61$, ${\upgamma }_{\mathrm{red}}=46$, ${\upgamma }_{\mathrm{white}}=8$), (**b**) after eight ticks (${\upgamma }_{\mathrm{blue}}=91$, ${\upgamma }_{\mathrm{red}}=16$, ${\upgamma }_{\mathrm{white}}=8$), and (**c**) after 29 ticks (${\upgamma }_{\mathrm{blue}}=102$, ${\upgamma }_{\mathrm{red}}=8$, ${\upgamma }_{\mathrm{white}}=5$)

Figure 4 shows three examples of our generated networks of how the opinion climate regarding four OLs changes over several ticks.

### Validation of the agent-based model

The validation of our agent-based model was carried out with a sensitivity analysis, i.e., a variation of different dimensions of input/output parameters, to determine how the parameter settings affect the behavior of the model. With the help of a sensitivity analysis, the different parameters of a model can be examined to increase the accuracy of the model, reduce the output variance, and simplify the model [90]. The various parameter spaces for our three scenarios to answer the three research questions are shown in Table 3. Here, the distribution of opinion leaders for the different opinion camps is particularly relevant. In combination with the total number of nodes including the number of opinion leaders in the network, we have adopted the divergent outcomes from the previous studies [2, 24] in our modeling and therefore implemented varying distributions of OLs in the network. We decided to consider 21 different distributions of opinion leaders, where in some distributions, a maximum of 20% of opinion leaders at 500 nodes and 10% of opinion leaders at 1000 nodes are investigated. Due to the variation in the distribution of OLs, the direct comparison between the ratio of opinion leaders from the different camps allows us to draw conclusions about the extent of influence on opinion formation in the network. Here, the two networks used can also ensure further conclusions about the topology in the network. Using the additional option of edge matching by means of a fixed number of randomized edges to the opinion leaders, we can clearly match the general definition of OLs, since they interact as a hub and are connected to many other agents in the network. The stepwise values for opinion leaders who take a discrediting opinion toward the other camp were chosen for the reason to be able to identify and compare possible tendencies of the opinion distribution more easily.

**Table 3:** Summary of agent-based modeling parameters

Parameter	Explanation	Description	Parameter space
V	Nodes	Tells the total number of nodes in the network (OLs and normal agents)	500, 1000
E	Edge adjustment	Provides an edge control functionality to determine whether additional edges of random nodes are connected to the OLs, thus strengthening the effect of the OLs in the network	True
Ψ	Discrediting	Provides a discrediting functionality for OLs	True, false
∇	Network topology	The modeling can be executed considering the two network structures of preferential attachment or Watts–Strogatz	Preferential attachment, Watts–Strogatz
${\sigma }_{blue}$, ${\sigma }_{red}$, ${\sigma }_{ambivalent}$	Number of blue, red, and ambivalent OLs	Represents the number of blue, red, and ambivalent OLs used in our modeling	[0, 1, 5, 12, 25, 50] [0, 1, 5, 12, 25, 50] [0, 1, 12, 20, 25, 50]
${\epsilon }_{blue}$, ${\epsilon }_{red}$, ${\epsilon }_{ambivalent}$	Number of random connected edges to OLs	If E equals true, then the number of edges from random agents is randomly connected to OLs. Allows the modification of network structures and settings	100, 100, 100
$-{\sigma }_{blue}$ $-{\sigma }_{red}$	Number of discrediting OLs	Indicates the number of discrediting OLs (parameter can be greater than the actual number of OLs ${\sigma }$ for the particular opinion camp)	[0] [0, 1, 5, 12, 25, 50]
${\lambda }_{blue}$ ${\lambda }_{red}$	Negative value for discrediting the other opinion	Represents a negative opinion value, which is in a range of –0.1 to –1. The higher the value, the higher is the negative influence in the network	0 [0, –0.2, –0.4, –0.6, –0.8, –1]

*OLs* opinion leaders

For each research question, we specified a different parameter space, so that different aspects such as baseline, ambivalent OLs, and discrediting OLs could be examined. In Appendix A (supplementary material), a more detailed subdivision of the parameter spaces has been included, which addresses the individual research questions

Furthermore, we applied the one-parameter-at-a-time (OAT) method to isolate each of the individual parameters and model it with different variations of other parameters. According to Lee et al. [91], this method increases the robustness of the model with repeated iterations. For our modeling, we set a value of 1000 for the iteration of each parameter space. As Waldherr & Wettstein [32] suggest, we determined an average result based on multiple iterations of the same parameter space. The variously applied parameter settings are not only intended to make the model realistic and to test it in this respect, but also to identify how the model behaves, for example at extreme values. We decided to set the number of agents in both networks based on previous studies that used agent-based modeling to investigate different social science theories such as the spiral of silence theory [10, 73], opinion leaders [15, 92], or general opinion dynamics [93, 94].

## Results and simulation experiments

To address our research questions, we generated an agent-based approach with 21 different distributions of OLs (see Table 3), varying with regard to the representation of the two opinion groups. Based on the different distributions, we can determine the ways in which the overall opinion climate is affected (e.g., the circumstances under which one OL gains a majority).

RQ1 asked how the opinion climate responds to a varying ratio of OLs who promote opposing stances on the same issue. To address RQ1, we first tested the scenario in which no OLs are present. Due to the fact that the modeling for the various parameter settings was performed on 1000 iterations, the results are considered on the averaged final state shown in Table 4. Here, on average, based on both network topologies, 5.48% of the nodes are ambivalent, 23.07% belong to the minority group, and slightly more than 71.44% are in the majority group. However, the subdivision of the two network topologies reveals differences in the distribution of the opinion climate. The most pronounced differences with regard to the applied network topology appear in the distribution of the ambivalent nodes when zero OLs are present in the network: while in the preferential attachment topology, 17.48% of nodes are ambivalent, this is the case for only 3.2% in the Watts–Strogatz network. Similarly, differences can be found in the minority group when there are no OLs: while this proportion is 31.53% in the preferential attachment network, it is 42.66% in the Watts–Strogatz network. The results for the majority group differ only slightly (50.99% preferential attachment, 54.14% Watts–Strogatz). However, the results for a strongly unbalanced distribution of OLs (i.e., 0 vs. 12 and 0 vs. 25, 0 vs. 50) demonstrate that in both network topologies, the majority group has a 93% win rate. Furthermore, when a critical mass of OLs is reached (at least 12 in each group), the fact that one opinion has twice as many OLs leads to further changes in the distribution of opinion climate.

**Table 4:** Results of the modeling with different opinion leader (OL) distribution and network topologies to evaluate the opinion distribution

OL distribution	Network topology	% Majority	% Minority	% Ambivalent
0	PA	50.99 CI [510.57, 51.4]	31.53 CI [31.21, 31.85]	17.48 CI [17.12, 17.85]
	WS	54.14 CI [54.34, 54.94]	42.66 CI [42.46, 42.86]	3.2 CI [3.13, 3.27]
0 vs. 1	PA	74.37 CI [74.08, 74.66]	18.01 CI [17.81, 18.22]	7.62 CI [7.48, 7.75]
	WS	75.64 CI [75.45, 75.83]	21.55 CI [21.37, 21.73]	2.81 CI [2.79, 2.84]
0 vs. 5	PA	90.12 CI [90.02, 90.21]	8.24 CI [8.17, 8.32]	1.64 CI [1.61, 1.67]
	WS	89.97 CI [89.88, 90.05]	8.39 CI [8.32, 8.46]	1.64 CI [1.62, 1.66]
0 vs. 12	PA	93.07 CI [93.02, 93.13]	6.14 CI [6.09, 6.18]	0.79 CI [0.78, 0.81]
	WS	92.8 CI [92.75, 92.86]	6.18 CI [6.14, 6.23]	1.01 CI [1, 1.03]
0 vs. 25	PA	93.24 CI [93.19, 93.29]	6.03 CI [5.98, 6.07]	0.74 CI [0.72, 0.75]
	WS	92.89 CI [92.84, 92.94]	6.13 CI [6.09, 6.18]	0.98 CI [0.97, 1]
0 vs. 50	PA	93.36 CI [93.31, 93.41]	5.97 CI [5.93, 6.01]	0.67 CI [0.66, 0.68]
	WS	92.99 CI [92.94, 93.04]	6.07 CI [6.03, 6.11]	0.94 CI [0.93, 0.96]
1 vs. 1	PA	48.5 CI [48.35, 48.66]	40.47 CI [40.33, 40.62]	11.02 CI [10.89, 11.16]
	WS	49.27 CI [49.18, 49.36]	44.07 CI [43.98, 44.16]	6.66 CI [6.59, 6.73]
1 vs. 5	PA	78.41 CI [78.32, 78.5]	16.78 CI [16.7, 16.86]	4.81 CI [4.77, 4.85]
	WS	78.63 CI [78.53, 78.73]	16.51 CI [16.42, 16.59]	4.86 CI [4.84, 4.89]
1 vs. 12	PA	88.09 CI [88.03, 88.16]	9.54 CI [9.48, 9.6]	2.37 CI [2.34, 2.39]
	WS	87.8 CI [87.72, 87.87]	9.42 CI [9.36, 9.48]	2.78 CI [2.76, 2.81]
1 vs. 25	PA	88.64 CI [88.58, 88.71]	9.18 CI [9.12, 9.23]	2.18 CI [2.16, 2.2]
	WS	88.16 CI [88.08, 88.23]	9.17 CI [9.11, 9.23]	2.68 CI [2.66, 2.7]
1 vs. 50	PA	89.04 CI [88.98, 89.1]	8.91 CI [8.86, 8.96]	2.05 CI [2.03, 2.07]
	WS	88.63 CI [88.56, 88.7]	8.88 CI [8.82, 8.94]	2.49 CI [2.52, 2.47]
5 vs. 5	PA	46.97 CI [46.9, 47.03]	43.75 CI [43.69, 43.81]	9.29 CI [9.22, 9.35]
	WS	46.78 CI [46.72, 46.84]	43.71 CI [43.65, 43.77]	9.51 CI [9.44, 9.57]
5 vs. 12	PA	67.47 CI [67.4, 67.54]	25.14 CI [25.07, 25.2]	7.39 CI [7.36, 7.43]
	WS	65.55 CI [65.48, 65.61]	26.05 CI [25.98, 26.13]	8.4 CI [8.36, 8.44]
5 vs. 25	PA	69.88 CI [69.81, 69.95]	23.33 CI [23.27, 23.4]	6.79 CI [6.76, 6.82]
	WS	67.02 CI [66.95, 67.1]	24.97 CI [24.89, 25.04]	8.01 CI [7.98, 8.04]
5 vs. 50	PA	71.65 CI [71.58, 71.73]	21.98 CI [21.92, 22.05]	6.36 CI [6.33, 6.4]
	WS	69 CI [68.91, 69.08]	23.52 CI [23.44, 23.6]	7.48 CI [7.45, 7.51]
12 vs. 12	PA	46.86 CI [46.8, 46.92]	43.7 CI [43.64, 43.76]	9.44 CI [9.38, 9.5]
	WS	46.17 CI [46.11, 46.22]	43.32 CI [43.26, 43.38]	10.51 CI [10.45, 10.57]
12 vs. 25	PA	49.05 CI [48.98, 49.11]	41.97 CI [41.9, 42.03]	8.99 CI [8.95, 9.03]
	WS	47.31 CI [47.26, 47.36]	42.65 CI [42.59, 42.7]	10.04 CI [10, 10.08]
12 vs. 50	PA	52.2 CI [52.13, 52.27]	39.37 CI [39.3, 39.43]	8.44 CI [8.4, 8.47]
	WS	50.11 CI [50.05, 50.18]	40.39 CI [40.32, 40.46]	9.49 CI [9.46, 9.53]
25 vs. 25	PA	47.68 CI [47.61, 47.75]	43.82 CI [43.75, 43.89]	8.5 CI [8.45, 8.55]
	WS	46.52 CI [46.46, 46.57]	43.82 CI [43.76, 43.87]	9.67 CI [9,61, 9.72]
25 vs. 50	PA	49.22 CI [49.16, 49.29]	42.73 CI [42.66, 42.79]	8.05 CI [8.02, 8.09]
	WS	48.23 CI [48.17, 48.28]	42.64 CI [42.58, 42.69]	9.14 CI [9.1, 9.17]
50 vs. 50	PA	48.06 CI [48, 48.13]	44.32 CI [44.26, 44.39]	7.61 CI [7.57, 7.66]
	WS	47.02 CI [46.97, 47.07]	44.37 CI [44.32, 44.42]	8.61 CI [8.56, 8.66]
Average	Both	71.44 CI [71.35, 71.54]	23.07 CI [22.99, 23.15]	5.48 CI [5.46, 5.50]

**Fig. 5:**
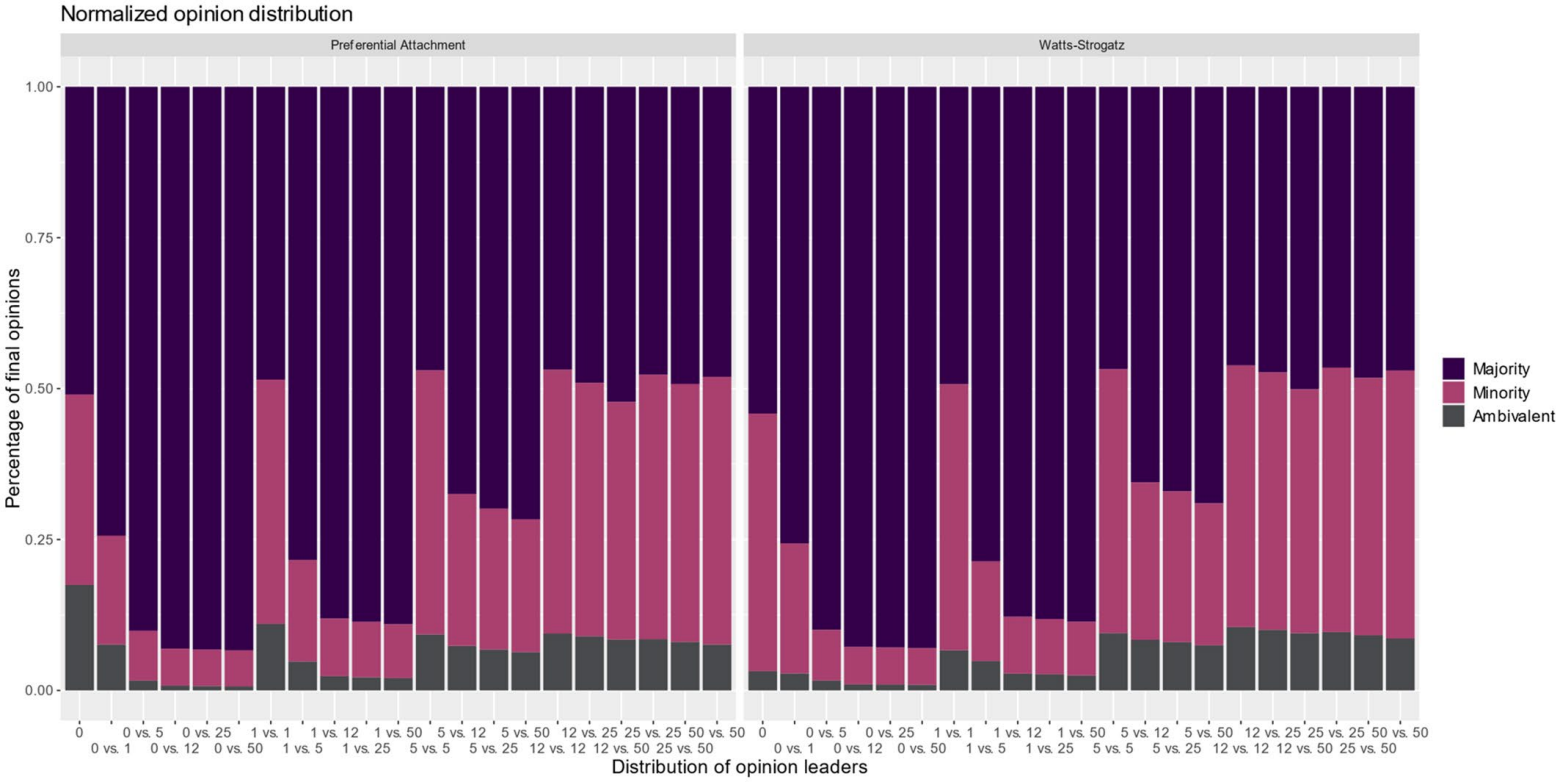
Normalized opinion distribution of 'majority,' 'minority,' and 'ambivalent' in relation to the distribution of opinion leaders

To make further statements about RQ1 with respect to the ambivalent nodes and changes in the opinion climate, we compared the initial and final shares of ambivalent nodes present in the network. This allows us to make a statement about the number of ambivalent nodes at the initialization of the network and at the end of the modeling. Interestingly, there were also differences in the ratios of the preferential attachment and Watts–Strogatz models when no OLs are present (see Fig. 5). In a state without OLs, the findings show that in a preferential attachment network, the number of ambivalent nodes in the network increases [from 9.49% CI (9.44%, 9.54%) to 17.48% CI (17.12%, 17.85%)], while in the Watts–Strogatz architecture, the number of ambivalent nodes decreases [from 9.46% CI (9.41%, 9.51%) to 3.20% CI (3.13%, 3.27%)]. However, this scenario without any OLs is relatively unlikely. Furthermore, it again appears that with a critical mass of OLs present in the network, the share of ambivalent opinions differs only by a minimal percentage. Our findings show that the number of ambivalent nodes remains comparatively constant over time, unless there is a strong imbalance in OLs (0:5, 0:12, 0:25 or 0:50).

**Fig. 6:**
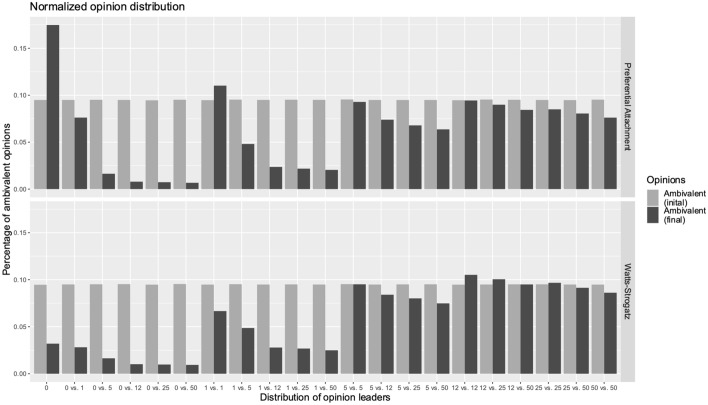
Results of the initial and final values of the ambivalent nodes in relation to the distribution of opinion leaders

As soon as an imbalance arises (0:12 or 0:25), the opinion camp with the higher number of OLs always wins. As soon as there are at least 12 OLs in both camps, the number of OLs does not matter, and the opinion climate becomes more balanced again. Figure 6 demonstrates the normalized opinion distribution of ambivalent agents at the initial state of the model and after the termination of the model and is clustered based on the two network topologies. The percentage distribution of ambivalent opinions can be seen on the y-axis, while the x-axis considers different distributions of OLs in the network.

**Fig. 7:**
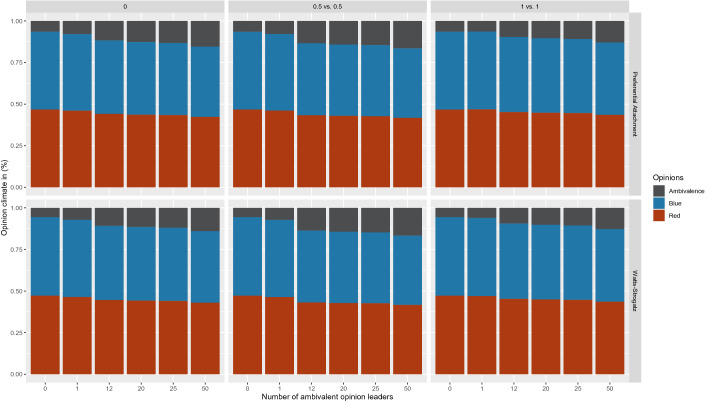
Impact of ambivalent opinion leaders on the climate of opinion in the network

RQ2 asked how the distribution of opinion climate responds to OLs who advocate in favor of one stance (univalent OLs) compared to ambivalent OLs. To this end, we ran a further model, this time including a varying number of ambivalent OLs (0, 1, 12, 20, 25, 50). This scenario thus includes OLs who advocate for the two opposing stances *r* and *b* with equal strength (i.e., *r* = *0.5* and *b* = *0.5 or r* = *1* and *b* = *1*). Furthermore, to increase the realism of the model, we decided to additionally include univalent OLs who represent the red or blue opinion camp. For this reason, we have included different numbers of OLs in the model over several iterations. Figure 7 shows a normalized opinion distribution based on the grouping of the ambivalent opinion values (0.5 vs. 0.5; 1 vs. 1), which include OLs and the resulting percentage distribution of the opinion climate (y-axis), separately for the two network topologies. The x-axis shows the number of OLs in the network (the results are averaged over multiple iterations).

When OLs are ambivalent, the results show that the number of agents with ambivalent opinions increases in the network. The difference between the two network topologies is very small and differs only marginally. The greatest effect is revealed when ambivalent moderate opinion strength with equally strong opinions of red and blue opinions (0.5 vs 0.5) is expressed in the network; an average percentage value of ambivalent agents of 11.40% CI [11.38%, 11.42%] after the end of the modeling results. Furthermore, in this context, it is noticeable that 1 OL and 12 OLs in the preferential attachment network have a percentage of 7.03% CI [6.98%, 7.08%] and 12.59% CI [12.52%, 12.65%] ambivalent agents, while 20, 25, and 50 OLs have a percentage of 13.32% CI [13.25%, 13.38%], 13.72% CI [13.66%, 13.79%], and 15.68% CI [15.62%, 15.74%] ambivalent agents. However, with a distribution of 1 versus 1, the average value of the climate of opinion in relation to the ambivalent nodes is lower at a value of 8.79% CI [8.78%, 8.80%]. Having said that, it is also shown that with an increase in OLs, the climate of opinion becomes more ambivalent. For example, the value is 10.87% CI [10.84%, 10.90%] for 12 OLs and rises to 14.11% CI [14.09%, 14.14%] for 50 OLs.

**Fig. 8:**
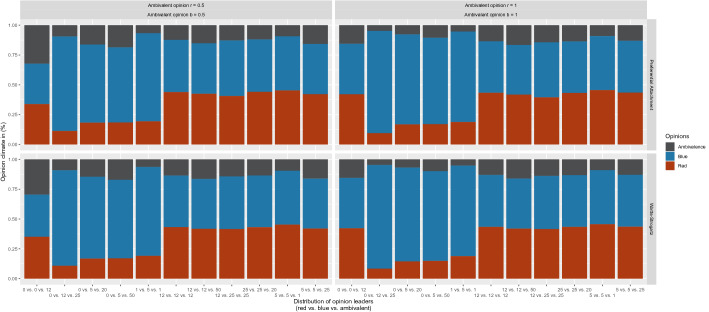
Impact of ambivalent (left: moderate; right: strong) and strongly ambivalent opinion leaders as well as the interconnection with other opinion leaders

In addition to the pure number of ambivalent OLs and their influence, the interaction between the different OLs is a key point to better understanding the dynamics of opinion formation. For this reason, we took a closer look at the climate of opinion on the different combinations of OLs in the network. More precisely, we examined the interaction and thus also the influence of the ambivalent OLs in relation to other OLs belonging to a certain opinion camp. In our model, as well as in the consideration of the results, we assume different distributions of the OLs (red OLs vs. blue OLs vs. ambivalent OLs). As an example, the following distribution would mean that there are no OLs in the red and blue opinion camps, but only 12 ambivalent OLs. Figure 8 shows this interaction between the different OLs of the opinion camps and the resulting distribution of opinion climate.

We exclusively focused on these ambivalent opinion values in the analysis, since, according to our definition, only the ambivalent OLs are considered to obtain further information.

The results show that in a distribution with only ambivalent OLs and a value of *r* = 0.5 and *b* = 0.5 for the opinions, the climate of opinion becomes balanced (35%). In this case, the climate of opinion of red and blue agents in the network is identical. With stronger opinion values of *r* = 1 and *b* = 1, ambivalent opinions in the network tend to decrease, and instead, red and blue opinions are more present in the network (42%). The results also show that if there is an imbalance of OLs in one opinion camp (0 vs. 12 vs. 25), it is not possible for those who are ambivalent OLs to create a balanced opinion climate. In Table 5, only a small percentage of ambivalent opinions (5–10%) and minority opinions (red, 8–11%) is shown, while the blue camp with the 12 OLs reaches a share of 79–87% in the network. The results in Table 5 show that, compared to the distribution of 12 versus 12 versus 12, there is still a relatively similar number of ambivalent agents. In particular, the distribution of agents in the blue opinion camp is then  43% for the preferential attachment and for the Watts–Strogatz model.

**Table 5:** Results of ambivalent opinion leaders interconnected with univalent opinion leaders

OL distribution	Network	Ambivalent value	% Blue	% Red	% Ambivalence
0 vs. 0 vs. 12	PA	*r* = 0.5, *b* = 0.5	33.84 CI [33.61, 34.08]	33.82 CI [33.58, 34.05]	32.34 CI [32.16, 32.52]
	WS	*r* = 0.5, *b* = 0.5	35.31 CI [35.14, 35.47]	35.11 CI [34.94, 35.28]	29.58 CI [29.46, 29.71]
	PA	*r* = 1, *b* = 1	42.35 CI [42.03, 42.68]	42.04 CI [41.72, 42.36]	15.61 CI [15.47, 15.75]
	WS	*r* = 1, *b* = 1	42.21 CI [42.02, 42.40]	42.24 CI [42.05, 42.43]	15.55 CI [15.45, 15.64]
0 vs. 5 vs. 20	PA	*r* = 0.5, *b* = 0.5	65.5 CI [65.34, 65.66]	18.25 CI [18.13, 18.38]	16.25 CI [16.16, 16.34]
	WS	*r* = 0.5, *b* = 0.5	68.64 CI [68.52, 68.76]	16.82 CI [16.72, 16.91]	14.54 CI [14.47, 14.62]
	PA	*r* = 1, *b* = 1	75.45 CI [75.29, 75.61]	16.82 CI [16.68, 16.96]	7.73 CI [7.67, 7.79]
	WS	*r* = 1, *b* = 1	78.59 CI [78.47, 78.72]	14.49 CI [14.38, 14.6]	6.92 CI [6.87, 6.97]
0 vs. 5 vs. 50	PA	*r* = 0.5, *b* = 0.5	62.98 CI [62.82, 63.14]	18.39 CI [18.26, 18.52]	18.63 CI [18.54, 18.72]
	WS	*r* = 0.5, *b* = 0.5	65.78 CI [65.65, 65.90]	17.06 CI [16.95, 17.16]	17.17 CI [17.09, 17.24]
	PA	*r* = 1, *b* = 1	72.39 CI [72.24, 72.54]	17.03 CI [16.89, 17.16]	10.58 CI [10.52, 10.64]
	WS	*r* = 1, *b* = 1	75.27 CI [75.15, 75.39]	14.86 CI [14.75, 14.96]	9.88 CI [9.83, 9.93]
0 vs. 12 vs. 25	PA	*r* = 0.5, *b* = 0.5	79.20 CI [79.08, 79.32]	11.29 CI [11.20, 11.38]	9.51 CI [9.44, 9.57]
	WS	*r* = 0.5, *b* = 0.5	80.01 CI [79.91, 80.11]	10.84 CI [10.76, 10.92]	9.15 CI [9.10, 9.20]
	PA	*r* = 1, *b* = 1	85.67 CI [85.57, 85.78]	9.51 CI [9.42, 9.59]	4.82 CI [4.78, 4.86]
	WS	*r* = 1, *b* = 1	86.9 CI [86.82, 86.99]	8.38 CI [8.31, 8.45]	4.72 CI [4.69, 4.75]
1 vs. 5 vs. 1	PA	*r* = 0.5, *b* = 0.5	73.8 CI [73.63, 73.96]	19.41 CI [19.27, 19.55]	6.79 CI [6.71., 6.87]
	WS	*r* = 0.5, *b* = 0.5	74.51 CI [74.40, 74.63]	19.1 CI [18.99, 19.2]	6.39 CI [6.33, 6.44]
	PA	*r* = 1, *b* = 1	75.75 CI [75.58, 75.92]	18.84 CI [18.70, 18.98]	5.41 CI [5.33, 5.48]
	WS	*r* = 1, *b* = 1	75.98 CI [75.86, 76.1]	18.83 CI [18.72, 18.94]	5.19 CI [5.15, 5.24]
5 vs. 5 vs. 1	PA	*r* = 0.5, *b* = 0.5	45.31 CI [45.19, 45.44]	45.29 CI [45.17, 45.42]	9.39 CI [9.32, 9.47]
	WS	*r* = 0.5, *b* = 0.5	45.13 CI [45.02, 45.24]	45.3 CI [45.19, 45.41]	9.57 CI [9.51, 9.64]
	PA	*r* = 1, *b* = 1	45.29 CI [45.16, 45.42]	45.53 CI [45.41, 45.66]	9.18 CI [9.10, 9.25]
	WS	*r* = 1, *b* = 1	45.32 CI [45.20, 45.43]	45.65 CI [45.54, 45.77]	9.03 CI [8.97, 9.09]
5 vs. 5 vs. 25	PA	*r* = 0.5, *b* = 0.5	42.08 CI [41.97, 42.19]	42.14 CI [42.03, 42.25]	15.78 CI [15.7, 15.86]
	WS	*r* = 0.5, *b* = 0.5	41.82 CI [41.71, 41.93]	42.04 CI [41.93, 42.15]	16.14 CI [16.07, 16.21]
	PA	*r* = 1, *b* = 1	43.34 CI [43.23, 43.46]	43.50 CI [43.38, 43.61]	13.16 CI [13.08, 13.24]
	WS	*r* = 1, *b* = 1	43.41 CI [43.30, 43.51]	43.6 CI [43.49, 43.7]	13 CI [12.93, 13.06]
12 vs. 12 vs. 12	PA	*r* = 0.5, *b* = 0.5	43.74 CI [43.63, 43.85]	43.90 CI [43.79, 44.02]	12.36 CI [12.29, 12.43]
	WS	*r* = 0.5, *b* = 0.5	43.21 CI [43.11, 43.30]	43.30 CI [43.08, 43.27]	13.62 CI [13.55, 13.68]
	PA	*r* = 1, *b* = 1	43.19 CI [43.08, 43.29]	43.30 CI [43.20, 43.41]	13.51 CI [13.44, 13.58]
	WS	*r* = 1, *b* = 1	43.51 CI [43.42, 43.6]	43.41 CI [43.32, 43.5]	13.08 CI [13.02, 13.14]
12 vs. 12 vs. 50	PA	*r* = 0.5, *b* = 0.5	42.32 CI [42.22, 42.43]	42.45 CI [42.35, 42.56]	15.22 CI [15.16, 15.29]
	WS	*r* = 0.5, *b* = 0.5	41.76 CI [41.66, 41.86]	41.82 CI [41.72, 41.92]	16.42 CI [16.36, 16.49]
	PA	*r* = 1, *b* = 1	41.64 CI [41.53, 41.74]	41.76 CI [41.66, 41.86]	16.61 CI [16.54, 16.67]
	WS	*r* = 1, *b* = 1	41.93 CI [41.84, 42.03]	41.94 CI [41.84, 42.03]	16.13 CI [16.07, 16.19]
12 vs. 25 vs. 25	PA	*r* = 0.5, *b* = 0.5	46.57 CI [46.45, 46.69]	40.61 CI [40.49, 40.72]	12.83 CI [12.77, 12.89]
	WS	*r* = 0.5, *b* = 0.5	44.16 CI [44.06, 44.26]	41.58 CI [41.48, 41.68]	14.26 CI [14.20, 14.32]
	PA	*r* = 1, *b* = 1	46.12 CI [46.01, 46.24]	39.5 CI [39.39, 39.61]	14.38 CI [14.31, 14.44]
	WS	*r* = 1, *b* = 1	44.53 CI [44.43, 44.62]	41.57 CI [41.48, 41.67]	13.9 CI [13.83, 13.96]
25 vs. 25 vs. 20	PA	*r* = 0.5, *b* = 0.5	43.92 CI [43.79, 44.05]	44.07 CI [43.93, 44.20]	12.01 CI [11.95, 12.07]
	WS	*r* = 0.5, *b* = 0.5	43.29 CI [43.20, 43.39]	43.18 CI [43.08, 43.27]	13.53 CI [13.47, 13.59]
	PA	*r* = 1, *b* = 1	43.13 CI [42.99, 43.26]	43.17 CI [43.03, 43.30]	13.71 CI [13.64, 13.77]
	WS	*r* = 1, *b* = 1	43.36 CI [43.26, 43.46]	43.43 CI [43.34, 43.53]	13.21 CI [13.14, 13.27]

*PA*, preferential attachment; *WS*, Watts–Strogatz

With regard to RQ2, it can be summarized that ambivalent OLs can ensure that the opinion climate concerning red and blue opinion is relatively stabilized and thus ultimately contribute to the existence of more ambivalent agents. Ambivalent OLs have the greatest influence when there are no other OLs from specific opinion camps in the network.

**Fig. 9:**
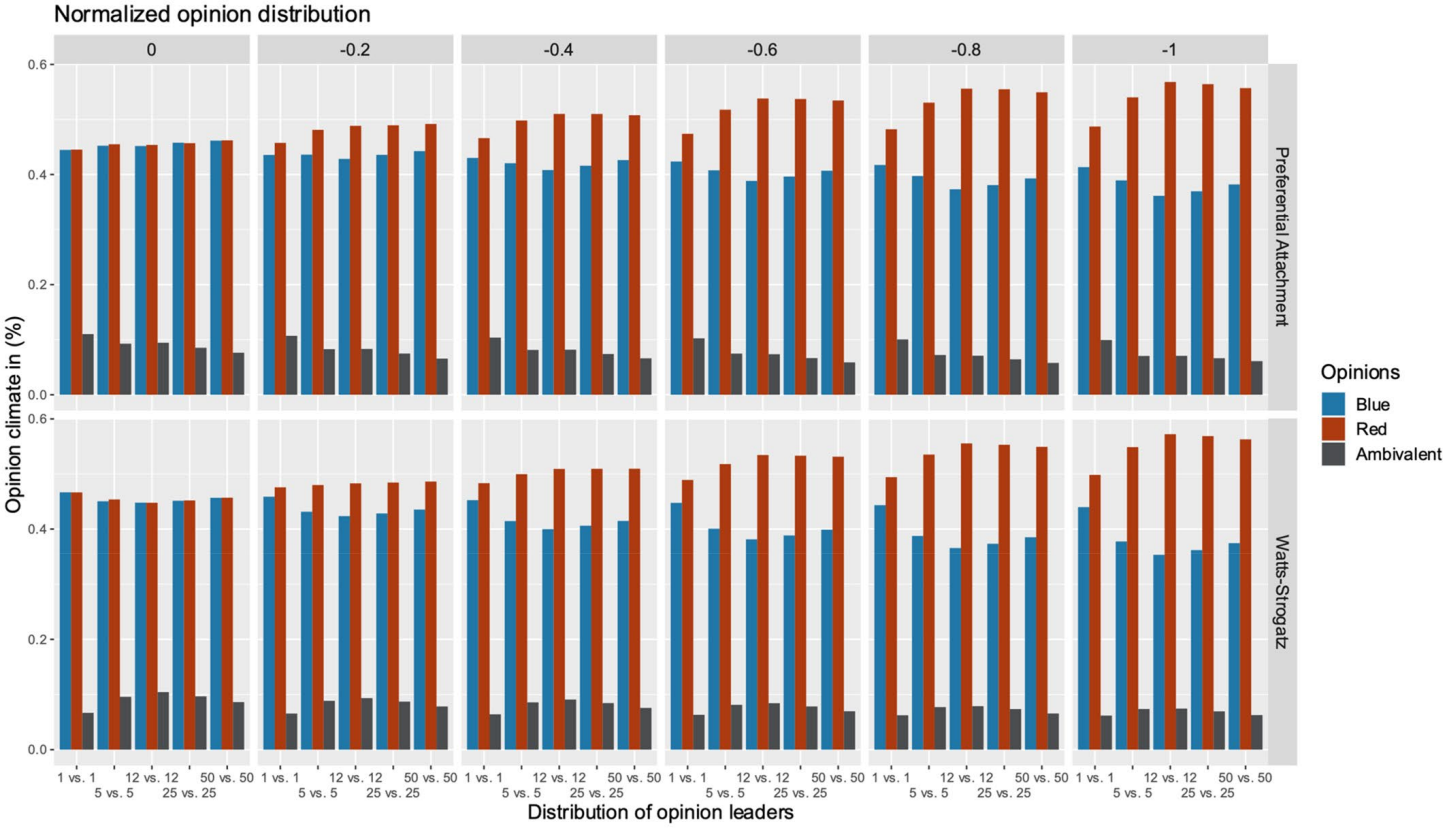
The effect of discrediting opinion leaders on the distribution of opinion climate

RQ 3 asked how the distribution of opinion climate responds to univalent OLs who discredit their opponent’s stance. To investigate the effect of discrediting OLs, we investigated five different distributions of OLs (1 vs. 1, 5 vs. 5, 12 vs.12, 25 vs. 25, and 50 vs. 50) in each camp *r* and *b*, since this scenario from the above findings leads to a stable opinion climate in which the two opinion camps are relatively equally distributed. Furthermore, we decided to increase the negative discredit value by –0.2 steps until the value of –1 was reached. In this scenario, we assumed that only OLs in the red camp may take on the role of discrediting the blue camp to have a clear comparative value. Regarding RQ3, the results in Fig. 9 show that the stronger the discrediting expression of an OL, the higher the probability that the opinion climate tips over in favor of the OL’s camp, in contrast to which supporters of the other party shrink to a minority. These findings can be found in both networks, the preferential attachment and the Watts–Strogatz network architecture. Compared to baseline (discreditation value: 0), where no discrediting takes place and the climate of opinion is symmetrical, the blue and red camps have across all distributions value of 49.26% CI [49.24%, 49.29%] for the preferential attachment and 49.46% CI [49.44%, 49.49%] for the Watts–Strogatz network. With an extreme discreditation value of –1, the size of the red opinion camp across all distributions has risen to 54.33% CI [54.23%, 54.43%] for the preferential attachment and 55.03% CI [54.92%, 55.13%] for the Watts–Strogatz network, while the blue camp has decreased to 38.31% CI [38.23%, 38.39%] and 38.13% CI [38.05%, 38.22%]. Interestingly, there were also differences in the ratios of ambivalent agents when increasing the discrediting value. The percentage of ambivalent opinions decreased in the preferential attachment model from 9.18% CI [9.16%, 9.20%] to 7.36% CI [7.33%, 7.39%] and in the Watts–Strogatz model from 8.99% CI [8.98%, 9.01%] to 6.84% CI [6.82%, 6.87%]. Thus, discrediting not only diminishes the discredited camp, but also the number of ambivalent nodes. To see a more detailed view about the different distributions, they can be seen in Table 6. Furthermore, the results have shown that the distribution of 12 vs. 12 OLs ensures that the opinion climate in the preferential attachment (51.90% CI [51.82%, 51.98%]), as well as in the Watts–Strogatz network (51.70% CI [51.62%, 51.79%]), develops most strongly in favor of the red camp across all negative discredit values.

**Table 6:** Results of the modeling with different opinion leader (*OL*) distribution and network topologies to evaluate the opinion distribution

OL distribution	Network	Rabble value	% Blue	% Red	% Ambivalence
1 vs. 1	PA	*r* = 0	44.46 CI [44.35, 44.58]	44.52 CI [44.4, 44.64]	11.02 CI [10.95, 11.09]
		*r* = − 0.2	43.55 CI [43.43, 43.67]	45.74 CI [45.62, 45.87]	10.7 CI [10.63, 10.77]
		*r* = − 0.4	43.01 CI [42.89, 43.13]	46.6 CI [46.48. 46.73]	10.38 CI [10.31, 10.45]
		*r* = − 0.6	42.36 CI [42.24, 42.48]	47.39 CI [47.26, 47.53]	10.25 CI [10.18, 10.31]
		*r* = − 0.8	41.73 CI [41.6, 41.86]	48.22 CI [48.08, 48.36]	10.05 CI [9.98, 10.12]
		*r* = − 1	41.34 CI [41.21, 41.47]	48.71 CI [48.57, 48.86]	9.94 CI [9.87, 10.01]
	WS	r = 0	46.67 CI [46.59, 46.74]	46.65 CI [46.58, 46.73]	6.68 CI [6.65, 6.71]
		*r* = − 0.2	45.87 CI [45.8, 45.95]	47.58 CI [47.5, 47.65]	6.55 CI [6.51, 6–58]
		*r* = − 0.4	45.25 CI [45.17, 45.33]	48.34 CI [48.25, 48.42]	6.41 CI [6.38, 6.44]
		*r* = − 0.6	44.76 CI [44.67, 44.84]	48.92 CI [48.83, 49.01]	6.32 CI [6.29, 6.36]
		*r* = − 0.8	44.34 CI [44.25, 44.43]	49.42 CI [49.33, 49.52]	6.24 CI [6.21, 6.27]
		r = − 1	43.98 CI [43.89, 44.08]	49.84 CI [49.74, 49.94]	6.18 CI [6.14, 6.21]
5 vs. 5	PA	*r* = 0	45.23 CI [45.18, 45.28]	45.5 CI [45.45, 45.55]	9.27 CI [9.24, 9.31]
		*r* = − 0.2	43.61 CI [43.55, 43.67]	48.11 CI [48.04, 48.19]	8.28 CI [8.24, 8.32]
		*r* = − 0.4	42.05 CI [41.96, 42.14]	49.81 CI [49.7, 49.92]	8.14 CI [8.1, 8.18]
		*r* = − 0.6	40.76 CI [40.65, 40.88]	51.77 CI [51.62, 51.92]	7.47 CI [7.42, 7.52]
		*r* = − 0.8	39.73 CI [39.59, 39.86]	53.05 CI [52.87, 53.23]	7.22 CI [7.17, 7.28]
		*r* = − 1	38.94 CI [38.78, 39.09]	54.02 CI [53.82, 54.21]	7.05 CI [6.99, 7.11]
	WS	*r* = 0	45.05 CI [45, 45.09]	45.38 CI [45.33, 45.42]	9.58 CI [9.54, 9.61]
		*r* = − 0.2	43.15 CI [43.09, 43.21]	48 CI [47.92, 48.07]	8.85 CI [8.82, 8.89]
		*r* = − 0.4	41.45 CI [41.36, 41.55]	49.97 CI [49.86, 50.09]	8.58 CI [8.54, 8.61]
		*r* = − 0.6	40.07 CI [39.94, 40.19]	51.8 CI [51.62, 51.96]	8.13 CI [8.09, 8.17]
		*r* = − 0.8	38.75 CI [38.6, 38.9]	53.53 CI [53.34, 53.72]	7.72 CI [7.67,7.77]
		*r* = − 1	37.75 CI [37.58, 37.92]	54.88 CI [54.66, 55.1]	7.37 CI [7.32, 7.43]
12 vs. 12	PA	*r* = 0	45.17 CI [45.13, 45.22]	45.38 CI [45.33,45.42]	9.45 CI [9.42, 9.48]
		*r* = − 0.2	42.84 CI [42.77, 42.91]	48.84 CI [48.75, 48.93]	8.32 CI [8.29, 8.36]
		*r* = − 0.4	40.81 CI [40.71, 40.92]	51.01 CI [50.87, 51.14]	8.18 CI [8.14, 8.22]
		*r* = − 0.6	38.84 CI [38.69, 38.99]	53.8 CI [53.61, 53.99]	7.36 CI [7.31, 7.42]
		*r* = − 0.8	37.32 CI [37.14, 37.5]	55.59 CI [55.36, 55.83]	7.09 CI [7.03, 7.15]
		*r* = − 1	36.13 CI [35.92, 36.34]	56.8 CI [56.55, 57.06]	7.07 CI [7.01, 7.12]
	WS	*r* = 0	44.79 CI [44.75, 44.84]	44.77 CI [44.73, 44.81]	10.43 CI [10.4, 10.46]
		*r* = − 0.2	42.35 CI [42.29, 42.42]	48.3 CI [48.21,48.39]	9.34 CI [9.31, 9.38]
		*r* = − 0.4	39.99 CI [39.88, 40.11]	50.92 CI [50.78, 51.06]	9.09 CI [9.05, 9.13]
		*r* = − 0.6	38.13 CI [37.98, 38.28]	53.45 CI [53.25, 53.64]	8.42 CI [8.38, 8.47]
		*r* = − 0.8	36.55 CI [36.37, 36.74]	55.56 CI [55.31, 55.8	7.89 CI [7.83, 7.95]
		*r* = − 1	35.32 CI [35.11, 35.54]	57.23 CI [56.95, 57.51]	7.45 CI [7.38, 7.52]
25 vs. 25	PA	*r* = 0	45.78 CI [45.72, 45.83]	45.69 CI [45.64, 45.75]	8.53 CI [8.51, 8.56]
		*r* = − 0.2	43.57 CI [43.5, 43.65]	48.94 CI [48.85, 49.03]	7.49 CI [7.45, 7.52]
		*r* = − 0.4	41.6 CI [41.49, 41.7]	51 CI [50.87, 51.13]	7.4 CI [7.37, 7.44]
		*r* = − 0.6	39.63 CI [39.48, 39.77]	53.72 CI [53.54, 53.9]	6.65 CI [6.61, 6.7]
		*r* = − 0.8	38.08 CI [37.9, 38.25]	55.49 CI [55.27, 55.71]	6.44 CI [6.39, 6.49]
		*r* = − 1	36.95 CI [36.75, 3715]	56.41 CI [56.17, 56.66]	6.63 CI [6.59, 6.68]
	WS	*r* = 0	45.14 CI [45.1, 45.18]	45.2 CI [45.16, 45.24]	9.66 CI [9.63, 9.69]
		*r* = − 0.2	42.83 CI [42.76, 42.89]	48.45 CI [48.37, 48.53]	8.72 CI [8.68, 8.75]
		*r* = − 0.4	40.61 CI [40.5, 40.72]	50.94 CI [50.81, 51.08]	8.45 CI [8.41, 8.48]
		*r* = − 0.6	38.84 CI [38.69, 38.98]	53.32 CI [53.14, 53.51]	7.84 CI [7.79, 7.89]
		*r* = − 0.8	37.33 CI [37.15, 37.51]	55.31 CI [55.08, 55.54]	7.36 CI [7.3, 7.41]
		*r* = − 1	36.17 CI [35.97,36.38]	56.88 CI [56.62, 57.14]	6.94 CI [6.88, 7.01]
50 vs. 50	PA	*r* = 0	46.16 CI [46.11, 46.21]	46.2 CI [46.15, 46.26]	7.64 CI [7.62, 7.66]
		*r* = − 0.2	44.25 CI [44.18, 44.31]	49.19 CI [49.1, 49.27]	6.57 CI [6.53, 6.6]
		*r* = − 0.4	42.62 CI [42.52, 42.71]	50.77 CI [50.65, 50.88]	6.62 CI [6.59, 6.65]
		*r* = − 0.6	40.68 CI [40.55, 40.81]	53.44 CI [53.27,53.61]	5.88 CI [5.83, 5.92]
		*r* = − 0.8	39.29 CI [39.13, 39.45]	54.93 CI [54.73, 54.13]	5.78 CI [5.73, 5.82]
		*r* = − 1	38.2 CI [38.02, 38.38]	55.69 CI [55.48, 55.91]	6.1 CI [6.07, 6.14]
	WS	*r* = 0	45.68 CI [45.64, 45.71]	45.7 CI [45.66, 45.74]	8.63 CI [8.6, 8.65]
		*r* = − 0.2	43.54 CI [43.48, 43.6]	48.63 CI [48.55, 48.7]	7.84 CI [7.81, 7.86]
		*r* = − 0.4	41.47 CI [41.37, 41.58]	50.96 CI [50.83, 51.08]	7.57 CI [7.54, 7.6]
		*r* = − 0.6	39.89 CI [39.76, 40.03]	53.14 CI [52.98, 53.31]	6.96 CI [6.92, 7.01]
		*r* = − 0.8	38.51 CI [38.35, 38.67]	54.93 CI [54.73, 55.14]	6.56 CI [6.51, 6.61]
		*r* = − 1	37.44 CI [37.25, 37.63]	56.29 CI [56.06, 56.53]	6.27 CI [6.21, 6.32]

*PA* preferential attachment model, *WS* Watts–Strogatz, *CI* 95% confidence interval

## Discussion

Applying agent-based modeling, this study investigated how OLs affect the opinion climate in online networks focusing on: (a) the impact of varying numbers of OLs in different opinion camps, (b) what influence ambivalent OLs exert in the network, and (c) how the opinion climate changes when OLs discredit the opposing opinion camp. Doing justice to the current state of knowledge in social psychology, these questions were examined in an opinion landscape in which individuals can have ambivalent opinions toward a certain issue. These scenarios are particularly relevant in the context of political communication, since OLs can spread their political stance (being univalent, ambivalent, or discrediting) in their network.

Addressing RQ1, we can state that an unequal distribution of OLs ensures that the opinion camp including the larger number of OLs—on average— “wins” over the opinion climate, i.e., the stance of the dominating OL is adopted by a majority of users in the network. Interestingly, however, even in the complete absence of OLs, the general opinion climate tips over time in favor of one side.

As our findings on RQ1 show, the numerically overrepresented group of OLs only dominates the general opinion climate when the other opinion camp is not represented by any OL. As soon as both reach a numerically critical mass (i.e., at least 12 OLs in each opinion camp), the advantage of the overrepresented camp disappears. This is in line with the previous findings that a few OLs can have a strong influence in the network and are responsible for the diffusion of opinions [21, 22, 95]. The finding can be explained by processes of complex contagion [96], i.e., the presence of multiple sources of influence within a complex social network structure. In such a structure, non-linear processes of influence often emerge. At the same time, the results show a "saturation effect," which reveals that at a certain point, an increasing number of OLs do not make a difference in how the opinion climate evolves. This observation may be explained by the idea of the "hard cores," that is, those who stick to their opinions in disregard of the external confirmations of other opinions, the conformity pressure exerted by others, and the potential social isolation that could be a consequence from being deviant from the majority [83, 97].

Our results also point to the boundaries of OL influence in complex and dynamically evolving social networks. These findings challenge the somewhat simplistic notion that OLs are primarily responsible for the formation of public opinion [1, 14], but rather put emphasis on the dynamically evolving social influence processes between “regular” users. In contrast to earlier findings (see [38]), our study shows that continuous increases in the number of OLs in a network only appear to impact public opinion under certain circumstances, i.e., when there is an extreme imbalance between OLs from opposing political camps. Furthermore, the impact of different shares of OLs who represent opposing stances appears to be dependent only to a limited extent on the network structure, with a somewhat higher amount of ambivalent users resulting in the preferential attachment network when no OLs are present at all (see [37]). Finally, network ambivalence (in terms of the share of attitudinally ambivalent users in the network) substantially decreases when there are only OLs advocating for one stance and remains largely unchanged when at least some OLs on each side are present. On one hand, this indicates that a network with an extreme imbalance of OLs from different opinion camps (e.g., within highly segregated networks) may foster further polarization over time [98], but on the other hand this shows that even a small number of opposing OLs may prevent a network from further segregation. These results are in line with the social psychological finding that consistently propagated minority views can have the potential to decrease majority influence [99] and emphasize that only some perseverant advocates of a minority stance can prevent a communication network from polarization.

In particular, when no opinion leader is represented, a difference between the two network topologies regarding the proportion of final ambivalent nodes was observable. A possible explanation of these results might be related to the strength of the weak ties in the different topologies [100]. Thus, it seems conceivable that the agents in the Watts–Strogatz network without opinion leaders and thus without a central hub are more weakly connected to each other in the network (weak ties), and thus, it is more difficult to come to a consensus of ambivalent opinions, since here the majority opinions of blue or red opinions have an advantage due to the ties. The preferential attachment model, on the other hand, has the characteristic that it has a heterogeneous attachment and has already formed hubs, which allows strong ties to emerge, where ambivalent nodes in a continuous loop can also hear other continuous opinions, even if these are associated with majority opinions.

Regarding RQ2, it can be concluded that the greater the number of ambivalent OLs in the network, the more likely it is that opposing opinion camps will be equally represented. Previous research documented that when OLs hold a particular stance, they can affect the network in favor of their stance [101, 102]. However, as addressed in the present study, it seems conceivable that OLs can also represent two-sidedness, e.g., by arguing simultaneously in favor and against a certain issue. When this situation occurs, it is more likely that the overall opinion climate is more balanced and also that more individuals in the network hold ambivalent attitudes themselves. Interestingly, the presence of moderately ambivalent OLs appears to lead to somewhat higher levels of network ambivalence compared to highly ambivalent OLs. While this appears to be independent of the specific network topology, moderate strength of OLs’ ambivalence may trigger just enough dynamic changes in the network to increase overall network ambivalence. Too many dynamic changes (caused by strongly ambivalent OLs), in contrast, lead to a somewhat lower network ambivalence. The degree of network ambivalence does not seem to increase with higher numbers of ambivalent OLs, but instead remains relatively stable when a certain threshold of OLs is reached. Again, this “saturation effect,” here on the ambivalence level, might be explainable by the notion that “hard cores” might believe in the correctness of their opinion and are less susceptible to influence by other network members [83, 97] These findings indicate that social influence may not only be exerted by key network actors promoting one particular stance, but also by those who promote ambivalence and thereby increase the “integrative complexity” of their network [51]. Our simulation implies that political discussion networks (e.g., on social media) that are characterized by a polarized opinion climate (with most individuals clearly favoring one of two opposing issue stances or attitudes) may already benefit when only some influential actors (e.g., journalists and politicians) advocate for balanced views. However, while these actors may foster depolarization by increasing ambivalent attitudes in the whole network, the low magnitude of effects suggests that their influence is comparatively limited. From a normative perspective, this insight is particularly interesting, since it points to potential boundaries of the impact that unbiased or mainstream media and public players have on public opinion (see Lau et al., 2017 [103]).

With regard to RQ3, the results showed that OLs publishing discrediting messages are more likely to win over the opinion climate than if they solely advocated in favor of their stance (without discrediting the other side). The findings of this study also imply that the more vigorously an OL discredits or argues against the opposing side, the more likely they are to shape majorities in a network. These findings reflect the ideals proposed by public sphere theory [104, 105]: when individuals promote their own stance and debate by “debunking” the arguments of the other side, they may succeed in reaching a consensus within a network.

In terms of theoretical implications for OL research, the present study allows insights into the distribution of OLs and what effect ambivalent and discrediting OLs can have on the climate of opinion in the network. As Newman [106] points out, understanding processes and behaviors in networks can help us understand complex phenomena that were previously difficult to explain. Our network analytic approach that modeled individual behavior as a function of external influence not only corroborates assumptions made by social psychology, predicting that one’s social ties that hold divergent attitudes can increase one’s personal attitudinal ambivalence [48, 50]. It also uncovered under which circumstances ambivalence can become a significant share within a network itself. While the ratio of opinion leaders and network topologies seem to be characteristics that shape the diffusion of ambivalence in a network, we also observed emergent phenomena such as the saturation effect of opinion leaders, potentially indicating that some actors are not susceptible to influence no matter how many hubs are present in a network. Implementation on a network level allows a consideration that is similar to social platforms such as Facebook or YouTube and thus takes into account the dynamic processes of the two-dimensional opinions of individuals, OLs, and their roles to measure their influence. Research on OLs usually focuses on their identification in networks [88, 107] and their specific characteristics [108]. Our results are intended to represent a theoretical basis for future research—integrating real-world data—to examine the relativizing effect of expressing ambivalent attitudes on the evolution of opinion climates. In particular, this research could serve to develop hypotheses in the field of political communication (e.g., in the context of election campaigns).

In terms of practical implications, results could play an important role in political and economic spheres. Political debates in online environments often appear to be clearly polarized and one-sided. Following the ideals of public sphere theory, it seems advisable to deal with the complexity, that is, the ambivalence of political attitudes and related arguments. Our research addresses this scenario and applies it to the existence of ambivalent OLs: based on our results, it appears that these ambivalent OLs can provide regulation of the opinion climate even when the proportions of OLs from two camps are different.

The findings regarding discrediting opinion leaders also reveal a practical implication in relation to contemporary journalism and news dissemination of information. The COVID-19 pandemic has once again highlighted the problematic presence of misinformation and its rapid spread on social media. Although the news reports on the scientific findings of the Coronavirus and their successes in the fight against the virus, misinformation still manages to get through to a portion of the population. Through social networks, discrediting opinion leaders who, for example, claim that the virus is harmless or that the government wants to control us with vaccination, can have an impact on their followers. To prevent this misinformation from influencing a wide proportion of the population, the government could implement countermeasures [109]. For example, journalists, public news houses, and influencers could serve as opinion leaders with a certain reach in their network and discredit counterarguments in order to fight misinformation.

Furthermore, the results can be used for specific business cases in the field of influencer marketing to promote product placements of opinion leaders more effectively and thus to identify and forecast the spread of opinions and future climates of opinion. This would allow companies to select which influencers are better suited for product placement to have an advantage over competing products and to promote them to the most appropriate networks.

In terms of implications for future research, our model can be extended to include other factors to analyze other theories besides OLs such as the spiral of silence theory, in which a person's opinion expression behavior is influenced by their environment. The integration of OLs in this model could also shed light on the point at which certain individuals no longer influence the opinions of others. Our work extends previous research by focusing on opinion leadership in networks by representing opinions two-dimensionally and, in particular, analyzing the functionality of ambivalent and discrediting OLs. As a key point for further research, we believe that our model can be carried out with real network data from social platforms to validate the results of our simulations [34].

## Limitations and further work

Our agent-based model solely represented two different camps of opinion, which can be compared, for example, to the political system in the United States, where Democrats and Republicans made up the lion's share of the opinion landscape. However, there are other political systems in which multiple parties exist, and therefore, more than two camps could be represented. Thus, future research could extend the existing binary modeling of opinions to include multiple opinions to study a more complex climate of opinion. In addition to the two applied network topologies, Watts–Strogatz and preferential attachment, other topologies could also be considered to make more specific statements on the role of network structure. In this context, we think that the investigation of the stochastic block models, which have the ability to form communities in graphs and to explicitly define their symmetric density, is particularly important. Using this model, findings showed that a low density in communities can lead to a reduction in the diversity of opinions [110]. In addition to the aspect of network topology, consideration could also be given to different centrality calculations, comparing the characteristics of OLs and the extent to which these change the climate of opinion. Besides the classic methods (closeness, betweenness) for identifying OLs, methods from game theoretical approaches might also be used.

Furthermore, future research could simulate the dynamics of opinion climates and the influence of OLs using real network data from platforms such as Facebook, YouTube, and Twitter. Here, a hybrid approach of sentiment analysis, network analysis, and agent-based modeling could be aimed at, in which a realistic representation of the opinion climate on different topics might be presented. One approach might be initially determining the opinions of users by means of an automatic sentiment analysis and filtering the individual topics on the basis of the thematic focuses. Next, the communication patterns (interactions) of the users with their calculated sentiment scores could be transformed into a network, which then serves as the basis for agent-based modeling. This could prove problematic in some cases, since the real data do not always fit 100% to the conditions of the network or vice versa. For example, although network data from social platforms show the flow of communication, a manual verification by humans would be required to ensure that opinions are exchanged on topics and establish whether they are for or against something. Likewise, the input to the models may require parameters that are not reflected in the real data, such as OLs who have been shown to influence individuals in the network. Finally, in our modeling, specific numbers of opinion leaders (0, 12, and 25) were included. Even though these were based on empirical knowledge of the prevalence of OLs on social networking sites, future research could make use of a more fine-grained distinction with smaller increments in the number of OLs.

## Conclusion

This study developed an agent-based model that deals with the phenomenon of opinion leadership and takes into account the ambivalence of different opinion camps on the basis of pertinent theoretical and empirical knowledge. The findings of this study show that OLs have an influence on the opinion climate, but that in particular, an extremely unequal distribution of OLs from different opinion camps leads to major adjustments (toward the dominating OL fraction) in the distribution of opinion climate on the network level. However, there appears to be a threshold value at which the imbalance of opposing OLs in the network no longer has any effect on the climate of opinion. This may indicate that in networks, social influence on the level of political opinions is subject to non-linear processes, where the global distribution of opinions changes in response to a critical number of opinion leaders from a political camp. To our knowledge, this is the first study that simulates processes of public deliberation and includes not only advocates of exclusively one viewpoint but also holders of ambivalent attitudes. We provide evidence that influential ambivalent players can increase ambivalence in individual users and counter processes of opinion polarization to a limited extent. Finally, we show that, when opinion leaders not only advocate for one side but also against the other side, the former opinion camp becomes more dominant within the network. Further research should model multiple attitude objects (i.e., more than one political issue) and systematically take the network structure and its dynamic changes into account (e.g., with regard to evolving clusters of like-minded users).

## Supplementary Information

Below is the link to the electronic supplementary material.

Supplementary file1 (DOCX 18 KB)
