# Supplementary material for: Two sides of the same leader: an agent-based model to analyze the effect of ambivalent opinion leaders in social networks
Source: J Comput Soc Sci. 2022 Apr 26;5(2):1159–205. doi: 10.1007/s42001-022-00161-z (PMC9039611; doi:10.1007/s42001-022-00161-z)
Supplement: Supplementary file 1 — Supplementary file1 (DOCX 18 KB) [file 42001_2022_161_MOESM1_ESM.docx]

**Appendix A**

| **Parameter** | **Explanation** | **Description** |
| --- | --- | --- |
| V | Nodes | Tells the total number of nodes in the network (OLs and normal agents) |
| E | Edge adjustment | Provides an edge control functionality to determine whether additional edges of random nodes are connected to the OLs, thus strengthening the effect of the OLs in the network |
| Ψ | Discrediting | Provides a discrediting functionality for OLs |
| ∇ | Network topology | The modelling can be executed considering the two network structures of preferential attachment or Watts–Strogatz |
| $\sigma_{blue}$,  $\sigma_{red}$,  $\sigma_{ambivalent}$ | Number of blue, red, and ambivalent OLs | Represents the number of blue, red, and ambivalent OLs used in our modeling |
| $\epsilon_{blue}$,  $\epsilon_{red}$,  $\epsilon_{ambivalent}$ | Number of random connected edges to OLs | If E equals true, then the number of edges from random agents is randomly connected to OLs. Allows the modification of network structures and settings |
| $-\sigma_{blue}$  $-\sigma_{red}$ | Number of discrediting OLs | Indicates the number of discrediting OLs (parameter can be greater than the actual number of OLs $\sigma$ for the particular opinion camp) |
| $\lambda_{blue}$  $\lambda_{red}$ | Negative value for discrediting the other opinion | Represents a negative opinion value, which is in a range of –0.1 to –1. The higher the value, the higher is the negative influence in the network |

Table 1. Description of parameter in our model

| **Parameter** | **RQ 1**  **Parameter space** | **RQ 2**  **Parameter space** | **RQ 3**  **Parameter space** |
| --- | --- | --- | --- |
| V | 500, 1000 | 1000 | 500, 1000 |
| E | True | True | True |
| Ψ | False | False | True |
| ∇ | Preferential attachment, Watts–Strogatz | Preferential attachment, Watts–Strogatz | Preferential attachment, Watts–Strogatz |
| $\sigma_{blue}$,  $\sigma_{red}$,  $\sigma_{ambivalent}$ | [0, 1, 5, 12, 25, 50]  [0, 1, 5, 12, 25, 50]  [0] | [0, 1, 5, 12, 25, 50]  [0, 1, 5, 12, 25, 50]  [0, 1, 12, 20, 25, 50] | [1, 5, 12, 25, 50]  [1, 5, 12, 25, 50]  [0] |
| $\epsilon_{blue}$,  $\epsilon_{red}$,  $\epsilon_{ambivalent}$ | 100,  100,  0 | 100,  100,  100 | 100,  100,  0 |
| $-\sigma_{blue}$  $-\sigma_{red}$ | 0  0 | 0  0 | [0]  [1, 5, 12, 25, 50] |
| $\lambda_{blue}$  $\lambda_{red}$ | 0  0 | 0  0 | 0  [0, –0.2, –0.4, –0.6, –0.8, –1] |
| *Iterations* | 1000 | 1000 | 1000 |
| *Total number of parameter space* | 144 | 3888 | 480 |

Table 2. Parameter space for the unique research questions
